# Elucidation of the pramanicin biosynthetic pathway reveals a DUF2306 family membrane protein involved in terminal epoxidation

**DOI:** 10.3389/fmicb.2026.1765828

**Published:** 2026-01-29

**Authors:** Yang-Le Gao, Wei Chen, Jing-Jing Zhang, Pei-Lin Li, Li Li, Hui Zhang

**Affiliations:** 1 Shengli Clinical Medical College of Fujian Medical University, Department of Breast Surgery, Fujian Provincial Hospital, Fuzhou University Affiliated Provincial Hospital, Fuzhou, China; 2 College of Life Sciences, Fujian Normal University, Fuzhou, China; 3 College of Bee Sciences and Biomedicine, Fujian Agriculture and Forestry University, Fuzhou, China

**Keywords:** biosynthesis, DUF2306, epoxidation, fungal natural products, *Macrophomina phaseolina*, PKS-NRPS, pramanicin

## Abstract

Pramanicin is a fungal metabolite with notable biological activities, including antifungal and anticancer properties. While its chemical synthesis has been achieved, its biosynthetic pathway has remained elusive. Here, we report the identification of the pramanicin biosynthetic gene cluster from the fungus *Macrophomina phaseolina*. Heterologous expression in *Aspergillus nidulans* demonstrated that the hybrid polyketide-nonribosomal (PKS-NRPS) enzyme PraA synthesizes a linear precursor that cyclizes to form pre-pramanicin. The flavin-dependent monooxygenase PraD and short-chain dehydrogenase/reductase PraB subsequently catalyze a hydroxylation and ketoreduction to yield pramanicin-A. Notably, we established that the final epoxidation step requires the PraC—a membrane-integrated protein of the previously uncharacterized DUF2306 family. This represents the first functional assignment of a DUF2306 family protein in natural product biosynthesis. Combinatorial expression and *in vivo* feeding experiments confirmed that PraC is essential for the formation of the bioactive epoxide moiety in pramanicin. Our work deciphers the biosynthetic pathway of a pharmaceutically relevant natural product, expands the enzymatic toolbox for synthetic biology by characterizing a novel family of membrane-associated biosynthetic enzymes.

## 1 Introduction

Fungi represent a prolific source of structurally complex natural products with diverse pharmacological activities, many of which have been developed into clinical therapeutics or serve as essential chemical probes (Atanasov et al., 2021; Newman and Cragg, 2020). The advent of inexpensive genome sequencing has revolutionized natural product discovery, revealing that the biosynthetic potential of fungi far exceeds the number of characterized metabolites (Irwin, 2021; Brakhage and Schroeckh, 2011; Rutledge and Challis, 2015; Wang and Mao, 2025). This discrepancy is largely attributed to the fact that many biosynthetic gene clusters (BGCs) remain transcriptionally silent under standard laboratory culture conditions, rendering their chemical products inaccessible through conventional fermentation-based approach (Keller, 2019; Eripogu Kiran et al., 2025). Consequently, genome mining coupled with heterologous expression has emerged as a powerful strategy to activate these cryptic pathways, enabling both the discovery of novel chemical entities and the elucidation of their biosynthetic logic (Chiang et al., 2023; Albarano et al., 2020; Chu et al., 2020; Scherlach and Hertweck, 2021; Chen et al., 2025).

Among the myriad of fungal metabolites, pramanicin stands out for its intriguing structure and significant bioactivity profile. First isolated from *Stagonospora* sp. ATCC 74235, pramanicin exhibits potent antifungal properties, selective cytotoxicity against human cancer cell lines such as Jurkat T-cells and colon carcinoma, and a unique ability to induce endothelial cell death via modulation of calcium permeability (Schwartz et al., 1994; Kutuk et al., 2005; Bodur et al., 2013; Kwan et al., 2001; Kwan et al., 2003). Structurally, it is characterized by a polar tetramic acid (or *γ*-lactam) pharmacophore derived from l-serine, fused to a hydrophobic polyketide chain that terminates in a synthetically challenging terminal epoxide moiety (Harrison et al., 1998; Harrison et al., 2000; Duspara et al., 1998). This epoxide is critical for its biological activity, as the non-epoxidized precursor (pramanicin-A) shows markedly reduced potency (Tan et al., 2015).

The intriguing bioactivity of pramanicin has spurred interest in its structural analogs, which share the core γ-lactam scaffold but exhibit a spectrum of biological activities due to variations in their polyketide side chains. Virgaricin, isolated from *Virgaria* sp., features a shorter polyketide side chain and exhibits significantly attenuated antimicrobial activity, underscoring the importance of side-chain length for antifungal potency (Ishii et al., 2012). In contrast, TMC-260, produced by *Acremonium kiliense*, possesses a polyketide chain similar in length to virgaricin but was identified as a specific inhibitor of Interleukin-4 (IL-4) signal transduction, indicating a remarkable shift from antimicrobial to immunomodulatory activity within this chemical family (Sakurai et al., 2003). Furthermore, the fungal strain *Aplosporella javeedii* was found to co-produce pramanicin A alongside a series of 11 new lactam derivatives, named aplosporellins A-K. Among these, pramanicin A itself demonstrated strong cytotoxicity against human lymphoma and leukemia cells by inducing caspase-3-mediated apoptosis (Gao et al., 2020). Most recently, new analogs virgaricins C and D were reported from *Apiospora* sp., displaying moderate antimalarial and cytotoxic activities, further expanding the known pharmacological scope of this compound family (Kimura et al., 2024). This collective body of work demonstrates that subtle modifications to the pramanicin scaffold can drastically alter biological specificity, yielding compounds with diverse therapeutic potential.

The significant biological profile of pramanicin has inspired sustained efforts in organic synthesis to access its complex architecture. A landmark achievement was reported in 1999 by Barrett and colleagues, who accomplished the first total synthesis of (+)-pramanicin, thereby unequivocally confirming its relative and absolute stereochemistry (Barrett et al., 1999). Despite these significant advances in chemical synthesis and biological evaluation, the genetic and enzymatic basis for pramanicin biosynthesis has remained an unsolved puzzle. Structure analyses strongly suggested a hybrid polyketide-nonribosomal peptide (PKS-NRPS) origin for its backbone, and the final epoxidation step was logically hypothesized to be catalyzed by canonical oxidative tailoring enzymes like cytochrome P450 monooxygenases or flavin-dependent monooxygenases (Woodcraft et al., 2023). This gap in knowledge regarding its biosynthetic pathway has hindered efforts to rationally engineer novel analogs with tailored properties or to understand the ecological context of its production.

**Figure 1:**
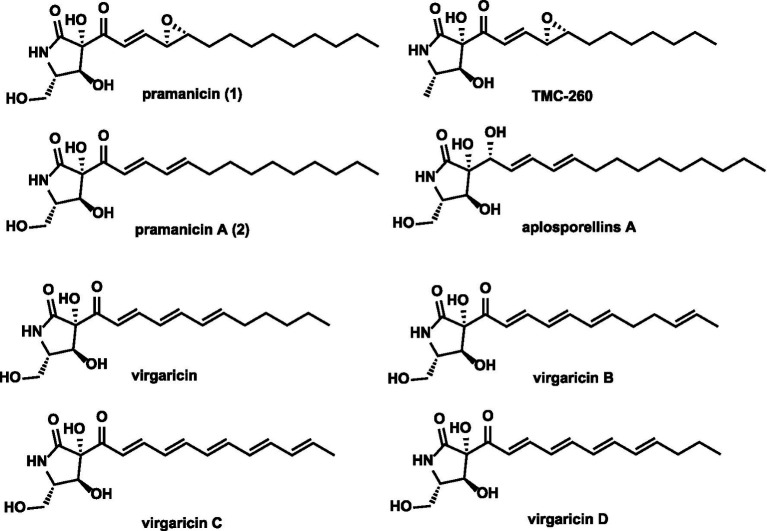
Structure of pramanicin and its related compounds.

The plant-pathogenic fungus *Macrophomina phaseolina* is a devastating agent of charcoal rot in over 500 plant species, including major economic crops such as soybean and cotton (Shirai and Eulgem, 2023). Beyond its agricultural impact, genomic analysis has revealed *M. phaseolina* to be a metabolically prolific organism, harboring a significant arsenal of biosynthetic gene clusters (BGCs) dedicated to secondary metabolism. The strain MS6, in particular, encodes more than 40 genes for polyketide synthases (PKSs), non-ribosomal peptide synthetases (NRPSs), and their hybrids, far exceeding the number of characterized metabolites from this fungus (Islam et al., 2012). Although in previous works, our group had identified macrophasetins (Yu et al., 2022) and macrollins (Gao et al., 2021) from MS6. The vast genomic potential indicates that a substantial reservoir of cryptic or silent BGCs still remains unexplored. Herein, we report the heterologous reconstitution and functional characterization of one mysterious cluster, which is dedicated to the biosynthesis of the known antifungal and cytotoxic metabolite pramanicin. Our work not only resolves the long-standing enigma of pramanicin biosynthesis but also, unexpectedly, assigns the crucial terminal epoxidation step to the first functionally characterized member of the DUF2306 protein family (Figure 1).

## 2 Materials and methods

### 2.1 General experimental procedures

Organic solvents for extraction and chromatography were of analytical grade or HPLC grade, as required. All chemical standards, unless otherwise specified, were purchased from Sigma-Aldrich (St. Louis, MO, USA). Enzymes for molecular biology were obtained from New England Biolabs (NEB; Ipswich, MA, USA) or Thermo Fisher Scientific (Waltham, MA, USA). Oligonucleotide primers were synthesized and purified by Tsingke Biotechnology Co., Ltd. (Beijing, China). PCR amplifications were performed using Phusion High-Fidelity DNA Polymerase (NEB) on a Bio-Rad T100 Thermal Cycler. Analytical LC–MS was conducted on a Thermo Scientific UltiMate 3,000 UHPLC system coupled to a Thermo Scientific LCQ Fleet ion trap mass spectrometer, using a Phenomenex Luna C18 column (3 μm, 2.0 × 150 mm). The mobile phase consisted of water (A) and acetonitrile (B), both containing 0.1% (v/v) formic acid, with a flow rate of 0.3 mL/min and a linear gradient from 5 to 95% B over 20 min. Preparative HPLC was performed on a Waters 2,545 system equipped with a 2,998 photodiode array detector and a preparative C18 column (XBridge BEH, 5 μm, 10 × 250 mm; Waters). NMR spectra, including ^1^H, ^13^C, COSY, HSQC, and HMBC, were recorded on a Bruker AVANCE III HD 600 MHz spectrometer at 25 °C, using methanol-d₄ or DMSO-d_6_ as solvents. Chemical shifts are reported in *δ* (ppm) relative to residual solvent signals. High-resolution mass spectrometry (HRMS) was performed on a Bruker microTOF-Q II mass spectrometer in positive electrospray ionization (ESI) mode. Optical rotations were measured with a Rudolph Research Analytical AUTOPOL V automatic polarimeter using a sodium lamp (*λ* = 589 nm) at 25 °C. Specific rotations ([*α*]D_T_) are given in units of 10^−1^ deg. cm^2^ g^−1^.

### 2.2 Strains and culture conditions

*Escherichia coli* DH5α was used for routine plasmid propagation and was cultivated in Luria Broth (LB) medium at 37 °C with appropriate antibiotics. *Saccharomyces cerevisiae* BJ5464-NpgA (MATα *ura3-52 his3-Δ200 leu2-Δ1 trp1 pep4: HIS3 prb1 Δ1.6 R can1 GAL*) was used for *in vivo* yeast homologous recombination and was cultured in YPD or synthetic drop-out (SD) medium as needed. The wild-type fungus *Macrophomina phaseolina* MS6 was maintained on potato dextrose agar (PDA) at 28 °C for genomic DNA extraction. The heterologous expression host *Aspergillus nidulans* A1145 (*ΔpyrG, ΔpyroA, ΔriboB*) was used for all heterologous expression experiments. *A. nidulans* was routinely maintained on CD medium (1% glucose, 5% 20 × nitrate salts, 0.1% trace elements, and 2% agar for solid media). For compound production, transformants were cultivated in liquid CD-ST medium (1 L: 20 g Starch, 20 g Peptone, 50 mL 20 × Nitrate salts, 1 mL Trace elements, pH 6.5) at 28 °C. Plasmids pYTU, pYTP, and pYTR were used as heterologous expression vectors (Li et al., 2016).

### 2.3 Bioinformatics analysis

Biosynthetic gene cluster (BGC) prediction was performed using antiSMASH 8.0^1^ with default parameters for fungal genomes. Domain architecture of PKS-NRPS was analyzed using the NCBI Conserved Domain Database (CDD) and PKS/NRPS analysis tools.^2^ Transmembrane helices were predicted with TMHMM 2.0.^3^ Homologous gene clusters were identified via BLASTP searches against the NCBI non-redundant (nr) and CAGECAT^4^ (van den Belt et al., 2023). Sequence alignments and identity/similarity calculations were performed using Clustal Omega and visualized with ESPript 3.0.

### 2.4 Plasmid construction and fungal transformation

All genes (*praA*, *praB*, *praC*, *praD*) were amplified from the genomic DNA of *M. phaseolina* MS6 using the Phusion High-Fidelity DNA Polymerase and gene-specific primers (listed in Supplementary Table S1). The large PKS-NRPS gene *praA* was amplified in three overlapping fragments of approximately 4 kb each using primer pairs UpraAF1/R1, UpraAF2/R2, and UpraAF3/R3 to ensure amplification fidelity. The three DNA fragments and pYTU digested with *Pac*I and *Swa*I were co-transformed into *S. cerevisiae* BJ5464-NpgA for assembly, leading to plasmid pYL135. The *praB*, *praD* and praC were also amplified using the primers of PpraBF/R, RpraDF/R and RpraCF/D, respectively, and cloned into pYTP and pYTR by yeast homologous recombination, leading to pYL136 and pYL137, pYL139. The praC also amplified with RpracF/R; the glaA promoter was amplified from pYTU with primers of glaA-pYTU-F/R; *praD* was obtained by PCR with primers of RpraDF/R. The three DNA fragments and linearized pYTR were co-transformed into *S. cerevisiae* BJ5464-NpgA for assembly, leading to plasmid pYL138. Yeast transformation was performed using Frozen-EZ Yeast Transformation II Kit (Zymo Research). The primers used for the heterologous expression are listed in Supplementary Table S1. The maps of pYL135-139 were shown in Supplementary Figure S1.

Protoplast transformation was performed using standard polyethylene glycol (PEG)-mediated methods (Li et al., 2018). Transformants were selected on CD agar plates lacking uracil, uridine, pyridoxine, or riboflavin, depending on the respective plasmid auxotrophic marker. The liquid CD-ST was used for the production of secondary metabolites in heterologous expression.

### 2.5 Fermentation, extraction, and compound purification

For the analysis of metabolites produced by heterologous expression, *A. nidulans* strains harboring the target *pra* genes were cultivated in liquid CD-ST medium at 28 °C with shaking at 220 rpm for 4 days. The entire culture was then extracted twice with an equal volume of ethyl acetate. The combined organic phases were dried under reduced pressure using a SpeedVac concentrator (Thermo Scientific). The resulting residue was dissolved in methanol, filtered through a 0.22 μm PTFE membrane, and subjected to LC–MS analysis.

LC–MS analyses were performed on a Thermo Scientific UltiMate 3,000 UHPLC system coupled to a Thermo Scientific LCQ Fleet ion trap mass spectrometer. Separation was achieved using a Phenomenex Luna C18 column (3 μm, 2.0 × 150 mm) maintained at 30 °C. The mobile phase consisted of (A) water and (B) acetonitrile, both containing 0.1% formic acid. A linear gradient was applied as follows: 5 to 95% B over 30 min, followed by holding at 95% B for 5 min, at a constant flow rate of 0.25 mL/min. MS detection was performed in positive electrospray ionization (ESI) mode.

For the isolation of compounds, a large-scale fermentation (12 L culture) was performed. The ethyl acetate extract from this fermentation was concentrated using a Buchi Rotavapor R-300 rotary evaporator. The crude extract was initially fractionated using a CombiFlash Rf+ purification system (Teledyne Isco) equipped with a reversed-phase C18 column (RediSep Rf Gold). Fractions were collected and monitored by analytical LC–MS. Those containing the target compounds were pooled and further purified by semi-preparative HPLC on a Phenomenex Luna C18 column (5 μm, 10 × 250 mm) using an optimized gradient of acetonitrile in water. The structures of the purified compounds were elucidated by comprehensive NMR spectroscopy. 1D and 2D NMR spectra, including 1H, 13C, COSY, HSQC, and HMBC, were acquired on a Bruker AVANCE III HD 600 MHz NMR spectrometer using methanol-*d_4_* or DMSO-*d_6_* as the solvent.

### 2.6 *In vivo* activity of PraC in *A. nidulans*

To validate the *in vivo* function of PraC, *A. nidulans* strains heterologously expressing *praC* under the control of the *glaA* promoter, along with empty vector controls, were pre-cultured in 50 mL of CD-ST medium for 48 h to reach the mid-logarithmic growth phase, ensuring high metabolic activity. Purified pramanicin-A was prepared as a 1 mg/mL stock solution in dimethyl sulfoxide (DMSO) to ensure solubility and sterility, and stored at −20 °C. Substrate concentration was titrated 50 ng/mL. After substrate addition, cultures were incubated for 24-h. Finally, the entire culture was extracted with ethyl acetate, concentrated, and redissolved in methanol for comprehensive LC–MS analysis.

### 2.7 Antifungal susceptibility testing

The *in vitro* antifungal activities of pramanicin, pramanicin-A, and pre-pramanicin were evaluated against the reference strains *Candida albicans* ATCC 90028, *Aspergillus fumigatus* ATCC 1022, *Aspergillus niger* ATCC 6275 and *Colletotrichum* spp. BCRC 35178. The Minimum Inhibitory Concentration (MIC) was determined using the broth microdilution method according to the standard reference procedures (Berkow Elizabeth et al., 2020).

## 3 Results

### 3.1 Identification and bioinformatics analysis of the pra cluster

**Figure 2:**
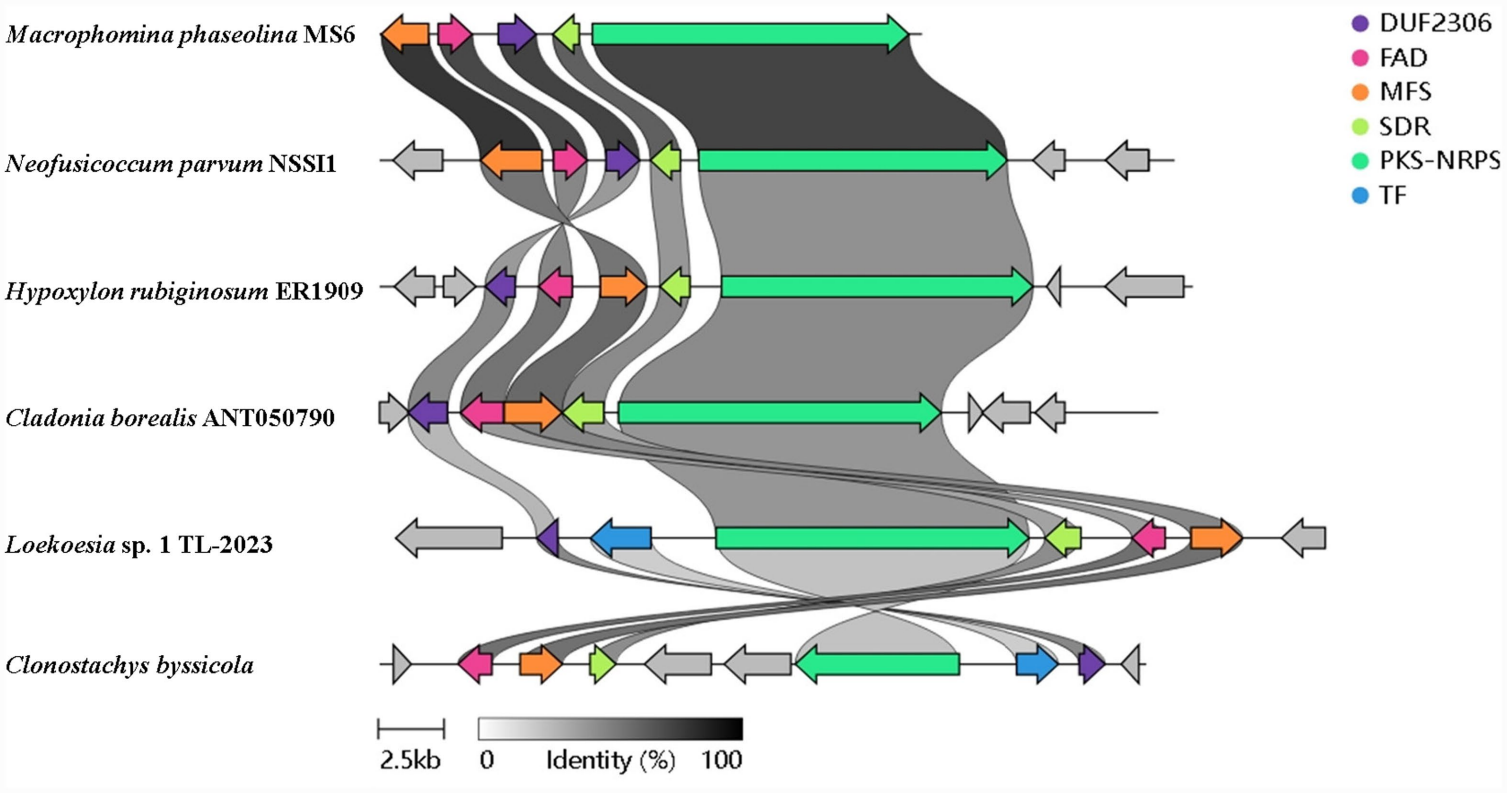
Bioinformation analysis of PRA cluster. Putative biosynthetic gene cluster of pramanicins in *Macrophomina phaseolina* and its similar clusters in other fungi; PKS-NRPS, polyketide synthase-nonribosomal peptide synthetase; SDR, short-chain dehydrogenase/reductase; DUF2306, domain of unknown function 2,306; FAD, flavin-dependent monooxygenase; MFS, major facilitator superfamily; TF: transcriptional regulatory factor. Genes within a gene cluster are color-coordinated. Similar genes found in multiple clusters have links drawn between and are shaded based on sequence identity. The scale bar indicates a gene length of 2.5 kb.

Analysis of the *M. phaseolina* MS6 genome led to the identification of a putative PKS-NRPS BGC, which we designated as the *pra* cluster (Figure 2). The core cluster consists of four genes: *praA*, encoding a highly reducing PKS-NRPS hybrid enzyme (3,940 aa) with domain architecture KS-AT-DH-ER-KR-ACP-C-A-PCP-R (KS, ketosynthase; AT, acyltransferase; DH, dehydratase; ER, enoylreductase; KR, ketoreductase; ACP, acyl carrier protein; C, condensation; A, adenylation; PCP, peptidyl carrier protein; R, reductase); *praB*, encoding a short-chain dehydrogenase/reductase (SDR, 302 aa); *praC*, encoding a predicted membrane protein (393 aa) with 5 predicted transmembrane helixes (TMHs) (Supplementary Figure S2), belonging to the functionally uncharacterized DUF2306 family; and *praD*, encoding a FAD-binding monooxygenase (432 aa) (Supplementary Table S2). Bioinformation analysis showed lots of fungi (eg. *Neofusicoccum parvum* and *Cladonia borealis*) have similar cluster with MS6 *pra* cluster. This indicate that the cluster is common in nature, maybe through Horizontal Gene Transfer (HGT).

### 3.2 Heterologous reconstruction of pramanicin biosynthesis and characterization of the products

**Figure 3:**
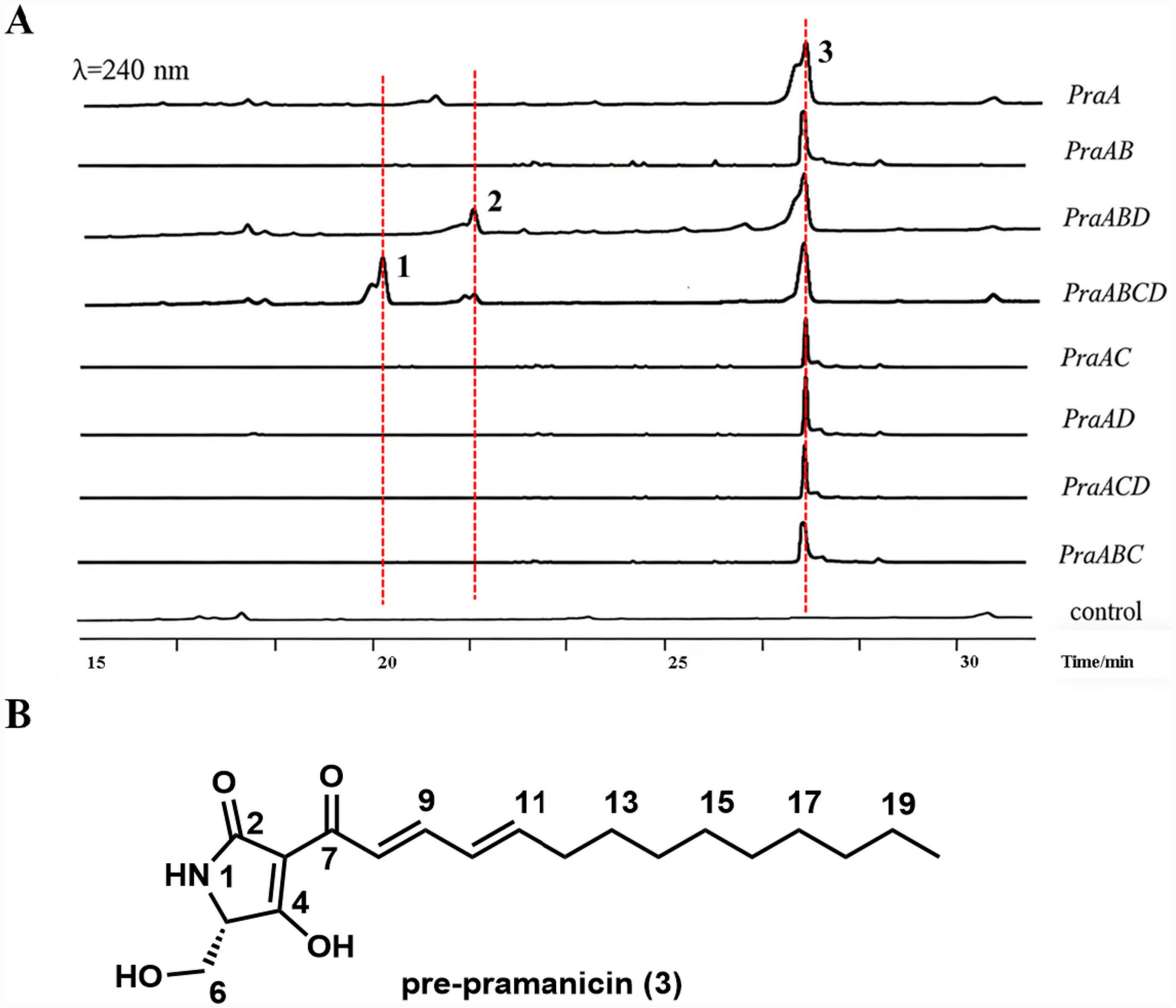
Genome mining of pramanicins by heterologous expression. **(A)** Heterologous expression of the *pra* cluster in *Aspergillus nidulans* A1145. HPLC profiles (*λ* = 240) of the extracts. **(B)** Chemical structures of pramanicins.

To confirm the function of the *pra* cluster, we employed a heterologous expression system in *A. nidulans* A1145. A series of plasmids (pYL135-pYL139) carrying different combinations of the *pra* genes under various inducible promoters were constructed (Supplementary Figure S1) and transformed into the host. Compared to the extract of the control strain harboring three empty vectors, one new metabolites (**1**) were accumulated in the extract of *A. nidulans* expressing *praABCD* (Figure 3A, trace iv). One new metabolites (**2**) were accumulated in the extract of *A. nidulans* expressing *praABD* (Figure 3A, trace iii). One new metabolites (**3**) were accumulated in the extract of *A. nidulans* expressing *praA* (Figure 3A, trace ii). The yields of pre-pramanicin (**3**), pramanicin-A (**2**), and pramanicin (**1**) from large-scale fermentation (12 L) were 5.2 mg/L, 1.9 mg/L, and 2.9 mg/L, respectively. Of which, **1** showed the molecular weight (MW) of 369, and **2** showed the MW of 353, **3** showed the MW of 335. These compounds were isolated and characterized by 1D and 2D NMR spectroscopy.

#### 3.2.1 Structural elucidation of 1

The structure of the final product **1**, was established through comprehensive NMR spectroscopy (Supplementary Table S3) and its optical rotation data: [*α*]25_D_ = −34.8° (*c* = 0.21 in MeOH) consistent with reported pramanicin ([*α*]25_D_ = −35° (*c* = 0.21 in MeOH)). The structure was thus unambiguously assigned as pramanicin according to literature data (Schwartz et al., 1994).

#### 3.2.2 Structural elucidation of 2

The ^1^H NMR spectrum of the intermediate **2** was similar to that of **1** but exhibited key differences indicative of the non-epoxidized precursor (Supplementary Table S4). The structure of **2** was further confirmed by 2D NMR (COSY, HSQC, HMBC), which delineated the complete polyene chain and its connection to the tetramic acid core consistent with pramanicin-A (Cadelis et al., 2021).

#### 3.2.3 Structural elucidation of the 3

Compound **3** ([M + H]^+^ = 336), produced by the strain expressing *praA* alone, was identified as the tetramic acid precursor prior to ketoreduction. Its ^1^H NMR spectrum lacked the diagnostic double bond signals in the polyketide chain observed in **2**, showing instead a set of aliphatic methylene signals. The ^13^C NMR spectrum confirmed the absence of the conjugated diene, with the corresponding carbons appearing as aliphatic methylenes. The molecular formula was determined to be C_19_H_29_NO_4_ by HRMS, corresponding to a reduced form of the PKS-NRPS product. 2D NMR analyses confirmed its structure as the linear precursor that undergoes spontaneous cyclization to form the tetramic acid ring system.

### 3.3 *In vivo* validation of PraC involvement in epoxidation

To validate the *in vivo* role of PraC in the epoxidation step, we fed the substrate pramanicin-A to *A. nidulans* strains heterologously expressing *praC* and analyzed the metabolic profiles using LC–MS.

**Figure 4:**
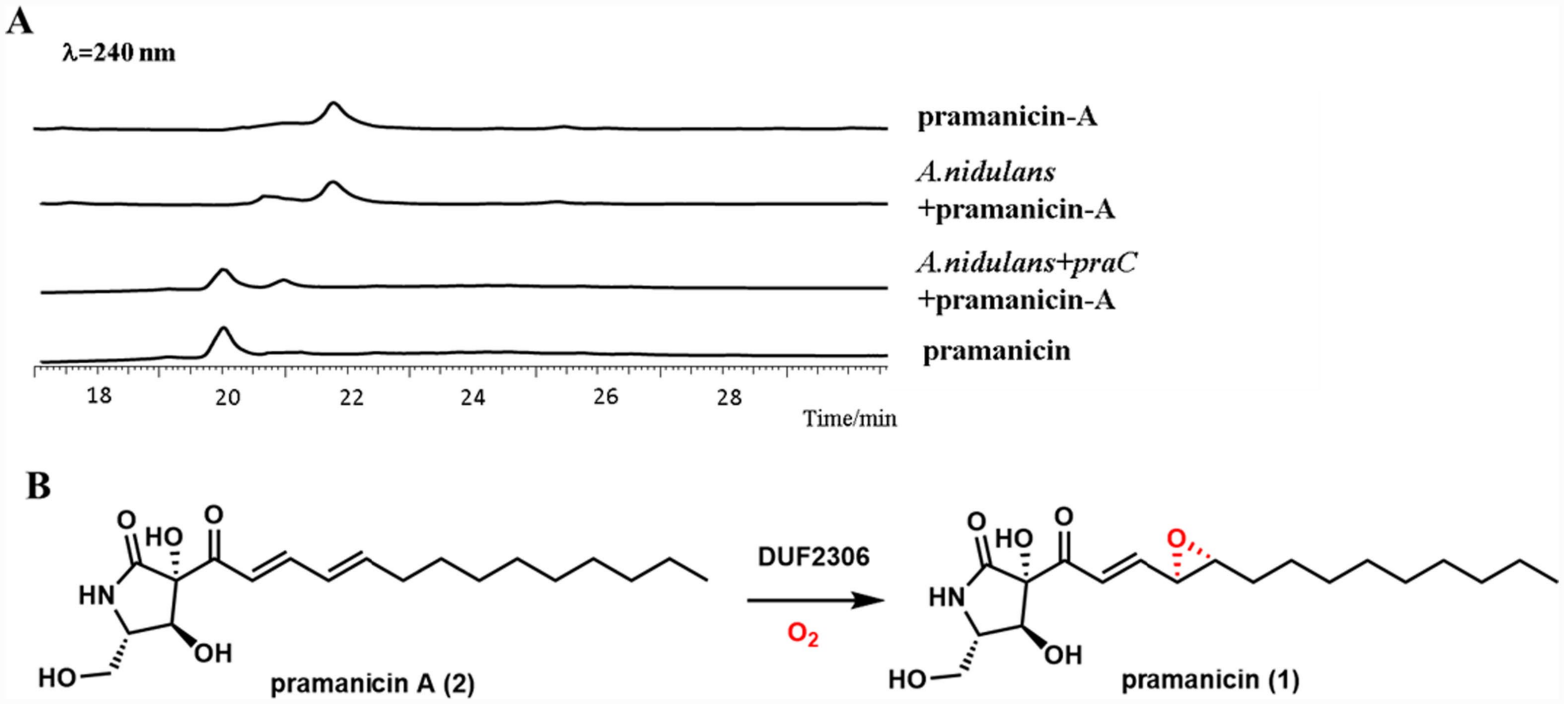
*In vivo* activity of PraC in *A. nidulans*. **(A)** Representative extracted chromatograms *λ* = 240 nm from *A. nidulans* strains. The strain expressing *praC* shows near-complete consumption of the substrate pramanicin-A and formation of the product, whereas the empty vector control does not. **(B)** PraC (DUF2306)-dependent epoxidation reaction.

Representative LC–MS chromatograms clearly demonstrated the specific conversion of pramanicin-A to pramanicin only in the experimental group (Figure 4). In the extract from the *A. nidulans* strain expressing *praC*, we observed a near-complete disappearance of the peak corresponding to pramanicin-A and the concomitant appearance of a new, prominent peak which matched the exact mass and chromatographic behavior of an authentic pramanicin standard. The observed mass shift of +16 Da is consistent with the addition of a single oxygen atom, confirming the epoxidation reaction.

Crucially, this conversion was entirely dependent on the presence of functional PraC. In the negative control strains transformed with an empty vector, the chromatogram was dominated by the substrate pramanicin-A peak, with no detectable formation of pramanicin. This result conclusively rules out the possibility that the epoxidation was catalyzed by endogenous enzymes of the *A. nidulans* host.

### 3.4 Biosynthesis of pramanicin

To elucidate the biosynthetic pathway of pramanicin and validate the function of each enzyme encoded by the *pra* cluster, we performed heterologous expression of different gene combinations in *A. nidulans* and analyzed the resulting metabolic profiles.

When the PKS-NRPS PraA was expressed alone in *A. nidulans*, the linear tetramic acid precursor **3** (m/z 336 [M + H]^+^) was detected as the major product (Figure 3A, trace i). This compound corresponds to the product released from the assembly line after Dieckmann cyclization, confirming that PraA is sufficient to synthesize the core backbone of pramanicin. The structure of **3** was elucidated by NMR spectroscopy (Supplementary Table S5, Supplementary Figures S16–22), revealing a linear polyketide chain attached to the tetramic acid moiety derived from L-serine.

Co-expression of *praA* with the short-chain dehydrogenase/reductase *praB* did not alter the product profile compared to *praA* alone, and compound **3** remained the dominant product. This suggested that the substrate for PraB is not the linear precursor **3**, but rather a later intermediate that is not accessible in this specific enzymatic context or requires further processing.

Strikingly, co-expression of *praA* with the flavin-dependent monooxygenase *praD* also failed to produce any new products, indicating that PraD alone cannot recognize or modify the linear precursor **3** to install the epoxide moiety.

However, when *praA*, *praB*, and *praD* were co-expressed together (*praABD*), a new compound **2** (m/z 354 [M + H]^+^) accumulated (Figure 3A, trace iii). This compound was isolated and characterized by extensive 1D and 2D NMR analyses as pramanicin-A (Supplementary Table S4, Supplementary Figures S9–15), the direct des-epoxy precursor of pramanicin. It was proposed that PraD catalyzes a hydroxylation on the linear precursor **3** (or a derivative thereof) then PraB catalyzes a ketoreduction to generate pramanicin-A, which subsequently serves as the substrate for the epoxidation reaction.

Crucially, the final epoxidation step to form bioactive pramanicin (compound **1**, m/z 370 [M + H]^+^) was only observed upon the introduction of all four biosynthetic genes, including *praC* (*praABCD*; Figure 3A, trace iv). This absolute requirement for PraC, a membrane-integrated DUF2306 family protein, for the formation of the epoxide is the most significant finding of this study.

**Figure 5:**
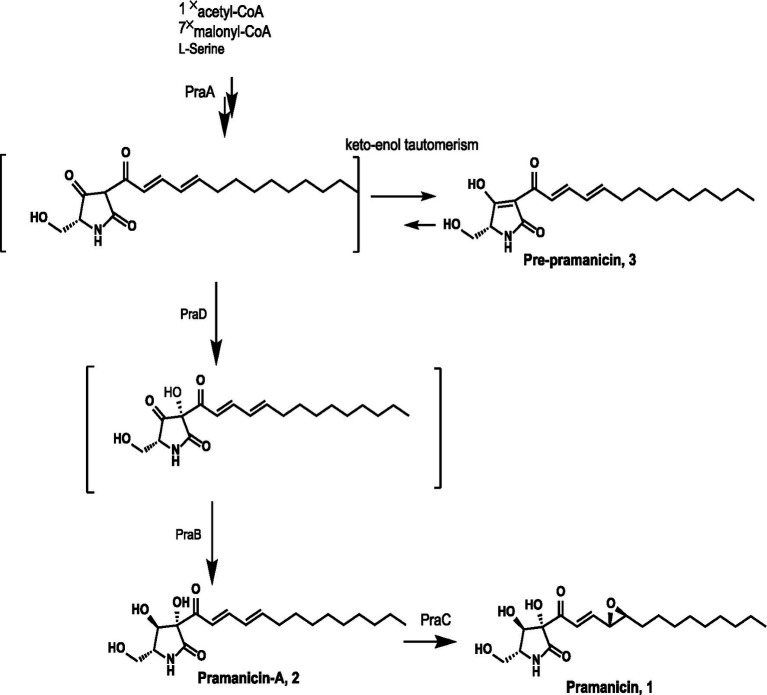
Proposed biosynthesis pathway of pramanicin.

Based on these findings, we propose the complete biosynthetic pathway of pramanicin as follows (Figure 5): The PKS-NRPS PraA assembles the polyketide chain from one acetyl-CoA and seven malonyl-CoA extender units, condenses it with l-serine, and releases the linear tetramic acid precursor **3** via Dieckmann cyclization. The FAD-binding monooxygenase PraD catalyzes a hydroxylation on the linear precursor **3** and the short-chain dehydrogenase/reductase PraB then catalyzes a key ketoreduction to yield pramanicin-A (**2**). Finally, the terminal epoxide is installed by the membrane protein PraC, producing the mature metabolite, pramanicin (**1**).

### 3.5 Antifungal bioactivity of pramanicins

The antifungal activities of the final metabolite pramanicin and its two immediate biosynthetic precursors, pramanicin-A and pre-pramanicin, were evaluated against the clinically significant yeast *Candida albicans* and the filamentous fungus *Aspergillus fumigatus*, *Aspergillus niger* and *Colletotrichum* spp. using the broth microdilution method. The minimum inhibitory concentration (MIC) values, summarized in Table 1, revealed a clear and profound structure–activity relationship (SAR).

**Table 1:** Antifungal susceptibility profiles of promanicins.

Organisms	Concentrations (μg/ml)		
Pathogenic fungi (MIC)	Pre-pramanicin	Pramanicin-A	Pramanicin
*Candida albicans* ATCC 90028	16	4	2
*Aspergillus fumigatus* ATCC 1022	32	16	4
*Aspergillus niger* ATCC 6275	16	8	2
*Colletotrichum* spp. BCRC 35178	32	8	4

Against the model yeast *Candida albicans*, pre-pramanicin showed moderate activity (MIC = 16 μg/mL). A 4-fold increase in potency was observed for pramanicin-A (MIC = 4 μg/mL), and the final metabolite pramanicin exhibited the strongest activity with an MIC of 2 μg/mL.

This trend of escalating potency with successive biosynthetic modifications was consistent across all tested fungi. For the two *Aspergillus* species, pramanicin was 4- to 8-fold more potent than pramanicin-A, and 8-fold more potent than pre-pramanicin against *Aspergillus niger*. Notably, pramanicin also demonstrated superior activity against the plant pathogen *Colletotrichum* spp., being 2- to 8-fold more potent than its precursors. In all cases, the fully elaborated pramanicin possessed the lowest MIC value, confirming it as the most active compound in the series.

## 4 Discussion

The elucidation of natural product biosynthetic pathways consistently serves as a fertile source of novel enzymatic paradigms (Scott and Piel, 2019). Our investigation yields two principal advances: the full genetic and enzymatic blueprint for pramanicin assembly, and, more importantly, the functional de-orphaning of a DUF2306 family protein. The finding that PraC, a membrane-integrated protein with no prior functional annotation, is indispensable for the terminal epoxidation challenges the prevailing paradigm that such reactions in fungi are solely the domain of cytochrome P450s or soluble monooxygenases (Thibodeaux et al., 2012).

While our *in vivo* data provide compelling evidence for PraC’s essential role, the precise biochemical mechanism remains an open and fascinating question. The membrane-associated nature of both PraC and its hydrophobic substrate, pramanicin-A, presents a formidable challenge for classical *in vitro* reconstitution. We posit that PraC may function as a unique epoxidase itself, perhaps utilizing a novel catalytic mechanism, or as an essential membrane scaffold that recruits and positions the substrate for oxidation by an as-yet-unidentified redox partner. Elucidating the exact mechanism—whether it involves direct oxygen activation or a facilitated reaction—will require the development of innovative assays with membrane mimics or proteoliposomes, a compelling direction for future structural and biochemical studies.

The implications of PraC’s function are profound and extend far beyond a single metabolite. The DUF2306 domain is ubiquitous in fungal and bacterial genomes. Our work effectively “de-orphans” this vast family and provides the first experimental evidence supporting its role in secondary metabolism. It is highly plausible that other DUF2306 members serve analogous, context-dependent functions as mem-brane-associated facilitators in the biosynthesis of other hydrophobic natural products. This discovery thus transforms DUF2306 from a genomic cipher into a valuable predictive marker for identifying BGCs that likely employ membrane-assisted tailoring steps, opening a new dimension for genome mining.

From a synthetic biology perspective, the functional expression of membrane-associated tailoring enzymes often constitutes a major bottleneck in the heterologous production of complex natural products. The identification and characterization of PraC-like facilitators provide a new genetic module to overcome this hurdle. The PraC system offers a plug-and-play module for synthetic biology strategies aimed at producing epoxidated derivatives of complex polyketides in heterologous hosts, where the membrane localization of the catalyst may prove advantageous for processing hydrophobic intermediates.

## 5 Conclusion

In summary, this study makes two pivotal contributions. First, we present the complete biosynthetic pathway of the antifungal and anticancer agent pramanicin, resolving its long-standing biosynthetic enigma. Second, and more significantly, we provide the first functional characterization for the widespread but obscure DUF2306 protein family, establishing its member PraC as an indispensable membrane-integrated protein required for a critical epoxidation reaction.

Our findings effectively demystify the biological role of the DUF2306 domain and propose its general function as a membrane-bound facilitator in the diversification of natural products. This breakthrough not only enriches the fundamental enzymatic repertoire known to natural product biosynthesis but also provides a fresh conceptual framework for future discovery and engineering. The PraC epoxidation system itself emerges as a valuable biocatalytic tool with significant potential for synthetic biology applications. Ultimately, this work underscores the immense, yet underexplored, potential of membrane-associated proteins in shaping structural diversity in nature’s small molecules.

## Data Availability

The original contributions presented in the study are included in the article/Supplementary material, further inquiries can be directed to the corresponding authors.

## Footnotes

1
https://antismash.secondarymetabolites.org/

2
https://nrps.igs.umaryland.edu/

3
https://services.healthtech.dtu.dk/service.php?TMHMM-2.0

4
https://cagecat.bioinformatics.nl
