# Supplementary material for: Elucidation of the pramanicin biosynthetic pathway reveals a DUF2306 family membrane protein involved in terminal epoxidation
Source: Front Microbiol. 2026 Jan 29;17:1765828. doi: 10.3389/fmicb.2026.1765828 (PMC12895699; doi:10.3389/fmicb.2026.1765828)
Supplement: Supplementary file 1 [file Data_Sheet_1.pdf]

Elucidation of the Pramanicin Biosynthetic Pathway Reveals a DUF2306 Family Membrane Protein Involved in Terminal Epoxidation

Yang-Le Gao[1, 2,3], Wei Chen[1], Jing-Jing Zhang[2], Pei-Lin Li[2], Li Li[2,*] , Hui Zhang[1, *]

1 Shengli Clinical Medical College of Fujian Medical University, Department of Breast Surgery, Fujian Provincial Hospital, Fuzhou University Affiliated Provincial Hospital, Fuzhou 350001, China

2 College of Life Sciences, Fujian Normal University, Fuzhou 350117, China

3 College of Bee Sciences and Biomedicine, Fujian Agriculture and Forestry University, Fuzhou 350002, China

*** Correspondence: lili@fjnu.edu.cn(LL); drzhanghui@fjmu.edu.cn (HZ)**

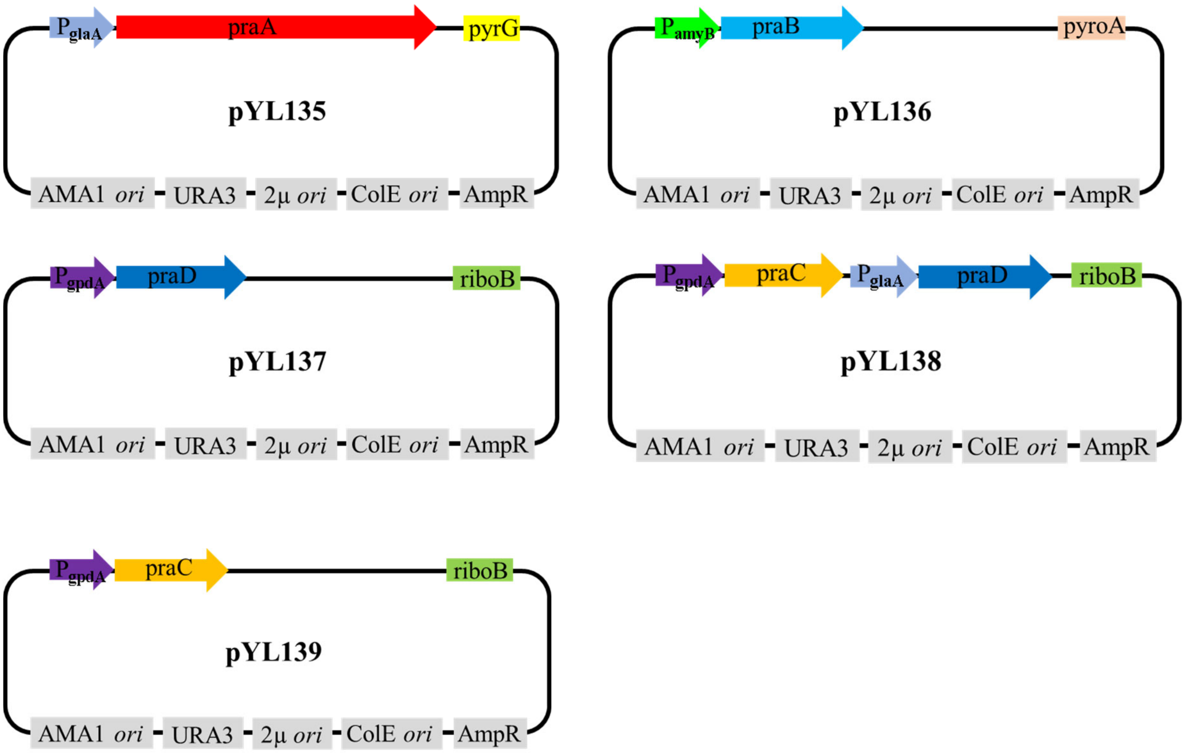

Supplementary Figure S1. Plasmids used in this study.

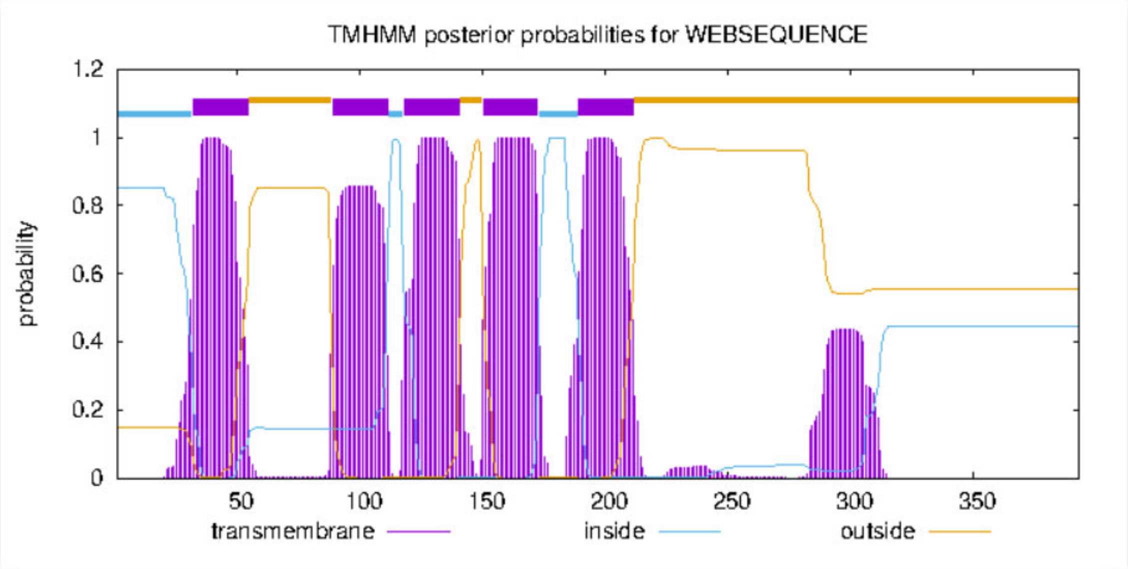

Supplementary Figure S2. Transmembrane helix prediction of PraC with TMHMN. PraC has 5 predicted TMHs.

Supplementary Table S1 Primers used in this study

Primer	Sequence (5’→3’)
UpraAF1	CTTCATCCCCAGCATCATTACACCTCAGCATTAATTAAATGAGC AGCACGCCATCAAGT
UpraAR1	CAGGTCGTATTTGGCTTCCTC
UpraAF2	TCTCAGGTGGCGCCTGTGAAG
UpraAR2	TGGATCGTACTGCAGATTCAG
UpraAF3	CTAGGCCAGATGGCCATCAAC
UpraAR3	AGTGGAGGACATACCCGTAATTTTCTGGGCATTTAAATGGGAT GAGAACGTGGCTAAGA
PpraBF	CTCCCTTCTCTGAACAATAAACCCCACAGAAGGCATTTATGAGC TTCTTCCAGGCACAG
PpraBR	GAGTGATGAGACCCAACAACCATGATACCAGGGGATTTCCTGT GTGGCCAGCTGAGTTG
RpraCF	ATTACCCCGCCACATAGACACATCTAAACATTAATTAAATGTCT CCCTCGCCCGACAGC
RpraCR	TAAAGGGTATCATCGAAAGGGAGTCATCCAATTTAAATGAGAT CATTGCTGAGGCCAAG
RpraDF	ATTACCCCGCCACATAGACACATCTAAACATTAATTAAATGTCT CCACGAATCCACGCA
RpraDR	TAAAGGGTATCATCGAAAGGGAGTCATCCAATTTAAATCCGCC ATTCTAGCCCCCGATG
RpraCDF1	ATTACCCCGCCACATAGACACATCTAAACATTAATTAAATGTCT CCCTCGCCCGACAGC
RpraCDR1	TCATAGGTCGCCAGGTACGACCAGTTCGGAAGATCAGGGAGAT CATTGCTGAGGCCAAG
glaA-pYTR-F	ACTCTTCCGTCAACTTGCTTGGCCTCAGCAATGATCTCCCTGAT CTTCCGAACTGGTCG
glaA-pYTR-R	ATGGCGATGCGCGGGCCTGCGTGGATTCGTGGAGACATTGCTG AGGTGTAATGATGCTG
RpraCDF2	AGCCTGAGCTTCATCCCCAGCATCATTACACCTCAGCAATGTCT CCACGAATCCACGCA
RpraCDR2	TAAAGGGTATCATCGAAAGGGAGTCATCCAATTTAAATCCGCC ATTCTAGCCCCCGATG

Supplementary Table S2 Bioinformatics analysis of *pra* gene cluster

Protein	Size (aa)	Proposed function	Homologs (identity)
PraA (EKG19785.1)	3940	PKS-NRPS KS-AT-DH-ER-KR-ACP-C-A-PCP-R	PytA (P9WEZ4.1) (46.92%)
PraB (EKG19784.1)	302	Short-chain dehydrogenase/reductase SDR	YanD (G3Y422.2) (39.60%)
PraC (EKG19783.1)	393	Predicted membrane protein (DUF2306)	(KZP25043.1) (26.13%)
PraD ((EKG19782.1)	432	putative monooxygenase FAD-binding protein	(KAB8360572.1) (53.41%)
Orf1 (EKG19781.1)	561	major facilitator superfamily domain （MFS）	(XP_033393654.1) (77.86%)

Supplementary Table S3 ^{1}H and ^{13}C NMR data for compound 1

position	pramanicin[1]		1	
	δH, mult (*J* in Hz)	δ_C	δH, mult (*J* in Hz)	δ_C
1				
2		174.94, C		174.97, C
3		88.09, C		88.11, C
4	4.15, d (7.2)	78.90, CH	4.18, d (7.3)	78.94, CH
5	3.47, ddd (7.2,5.4,2.8)	60.26, CH	3.47, ddt (9.0,6.4,3.2)	60.29, CH
6	3.79，dd (11.7,2.8)<br>3.55，dd (11.7,5.4)	61.99, CH2	3.82, dd (11.7,2.9)<br>3.58, dd (11.6,5.4)	62.03, CH_2
7		197.88, C		197.93, C
8	7.05，dd (15.6, 0.7)	127.84, CH	7.08, d (15.6)	127.88, CH
9	6.64，dd (15.6, 7.0)	145.13, CH	6.67, dd (15.6, 7.1)	145.16, C
10	3.38，dd (7.5, 1.9)	57.79, CH	3.35, d (1.7)	57.79, CH
11	2.93, ddd (6.2, 4.9, 1.9)	62.88, CH	2.96, td (5.7, 5.0, 2.0)	62.90, CH
12	1.60, m	33.05, CH_2	1.66, m<br>1.58, m	33.04, CH_2
13	1.45, m	26.97, CH_2	1.49, m	26.95, CH_2
14-18	1.29, bs	30.05, CH_2	1.33, m	33.04, CH_2
14-18	1.29, bs	30.66, CH_2	1.33, m	30.65, CH_2
14-18	1.29, bs	30.62, CH_2	1.33, m	30.63, CH_2
14-18	1.29, bs	30.51, CH_2	1.33, m	30.50, CH_2
14-18	1.29, bs	30.44, CH_2	1.33, m	30.42, CH_2
19	1.29, bs	23.73, CH_2	1.33, m	23.71, CH_2
20	0.89, t (6.8)	14.43, CH_3	0.92, t (7.0)	14.42, CH_3

Supplementary Table S4 ^1H and ^{13}C NMR data for compound 2

position	Pramanicin-A[2]		2	
	δH, mult (*J* in Hz)	δ_C	δH, mult (*J* in Hz)	δ_C
1				
2		175.26, C		175.31, C
3		88.07, C		88.08, C
4	4.14, d (7.0)	79.01, CH	4.15, d (7.2)	78.95, CH
5	3.51, ddd (7.5, 5.5, 2.5)	60.39, CH	3.52, ddd (7.2, 5.4, 2.7)	60.34, CH
6	3.80, dd (11.0,2.5)	62.20, CH_2	3.81, dd (11.5,2.7)	62.09, CH_2
	3.55, dd (11.0,5.5)		3.56, dd (11.5,5.5)	
7		198.70, C		198.73, C
8	6.72, d (15.0)	124.25, CH	6.74, d (15.2)	124.22, CH
9	7.27, ddm (15, 10)	145.53, CH	7.29, m	145.50, CH
10	6.2-6.3, m	130.48, CH	6.31, m, overlap	130.49, CH
11	6.2-6.3, m	148.58, CH	6.31, m, overlap	148.61, CH
12	2.19-2.22, m	34.20, CH_2	2.22, td (7.3, 5.4)	34.22, CH_2
13	1.44, q (7.5)	29.82, CH_2	1.45, m	29.84, CH_2
14-18	1.29, m	30.29, CH_2	1.30, m	30.32, CH_2
14-18	1.29, m	30.63, CH_2	1.30, m	30.66, CH_2
14-18	1.29, m	30.53, CH_2	1.30, m	30.56, CH_2
14-18	1.29, m	30.40, CH_2	1.30, m	30.43, CH_2
18	1.29, m	33.02, CH_2	1.30, m	33.05, CH_2
19	1.29, m	23.69, CH_2	1.30, m	23.72, CH_2
20	0.89, t (7.0)	14.40, CH_3	0.90, t (7.0)	14.43, CH_3

Supplementary Table S5 ^{1}H and ^{13}C NMR data for compound 3

position	δ_H, mult (*J* in Hz)	δ_C	COSY
1			
2		175.25, C	
3		101.03, C	
4		172.73, C	
5	3.84, s	63.97, CH2	H6
6	3.61, qd (11.3, 3.4)	60.48, CH	
7		194.25, C	
8	6.99. d (13.4)	119.33, CH	H9
9	7.39, d (15.8)	143.98, CH	H10
10	6.40, m, overlap	129.37, CH	
11	6.40, m, overlap	147.80, CH	H12
12	2.19, q (5.3, 4.2)	32.65, CH2	H13
13	1.40, q (7.0)	28.09, CH2	H14
14-18	1.25, m	28.95, CH2	
14-18	1.25, m	28.84, CH2	
14-18	1.25, m	28.70, CH2	
14-18	1.25, m	28.64, CH2	
18	1.25, m	31.30, CH2	
19	1.25, m	22.12, CH2	H20
20	0.85, t (6.9)	13.97, CH3	

Figure S3. ^{1}H NMR spectrum (150 MHz) of compound **1** in MeOD-*d*4

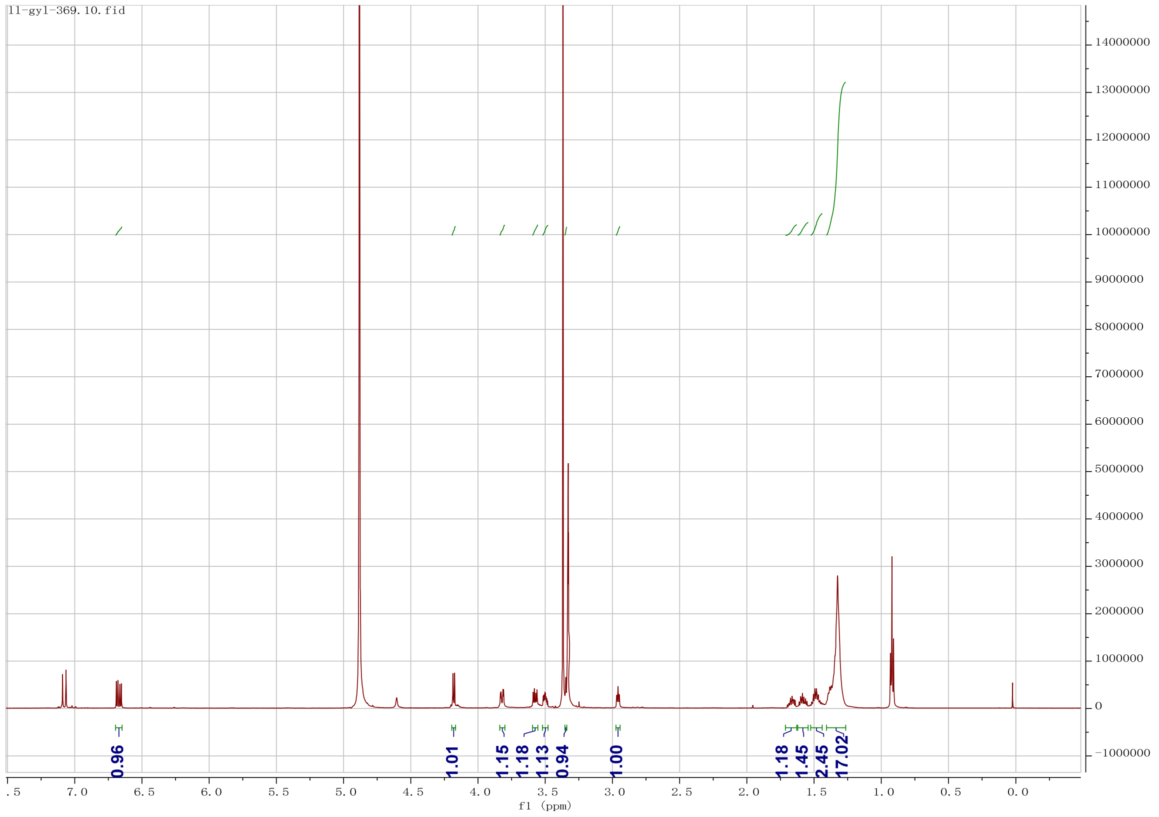

Figure S4. ^{13}C NMR spectrum (600 MHz) of compound **1** in MeOD-*d*4.

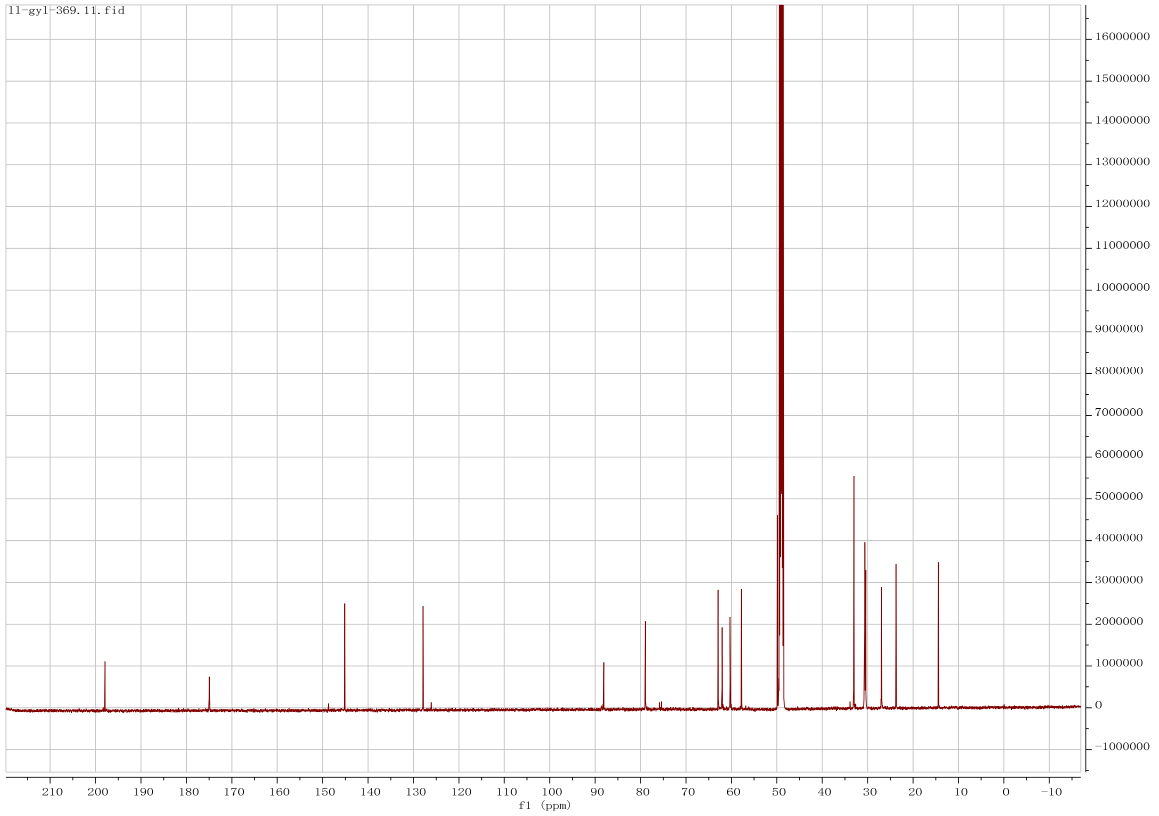

Figure S5. DEPT135° spectrum (150 MHz) of compound **1** in MeOD-d_4.

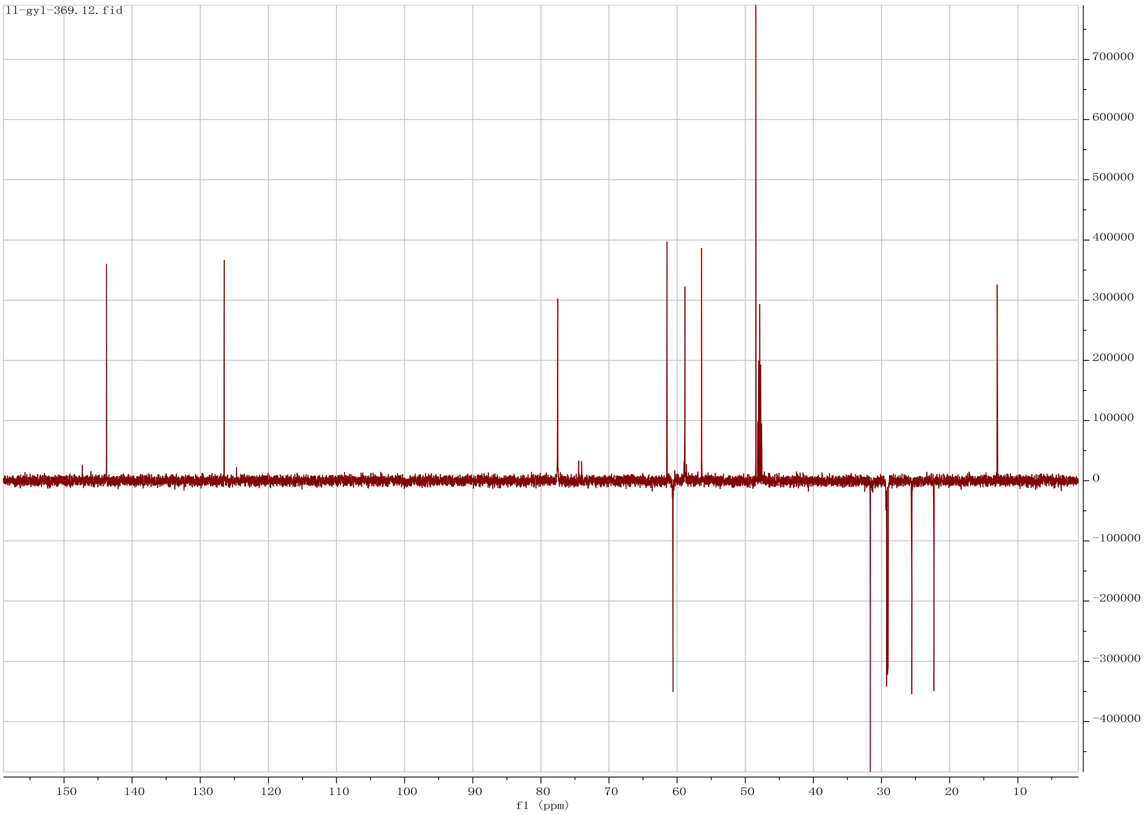

Figure S6. ^{1}H-^{1}H COSY NMR spectrum of compound **1** in MeOD-d_4

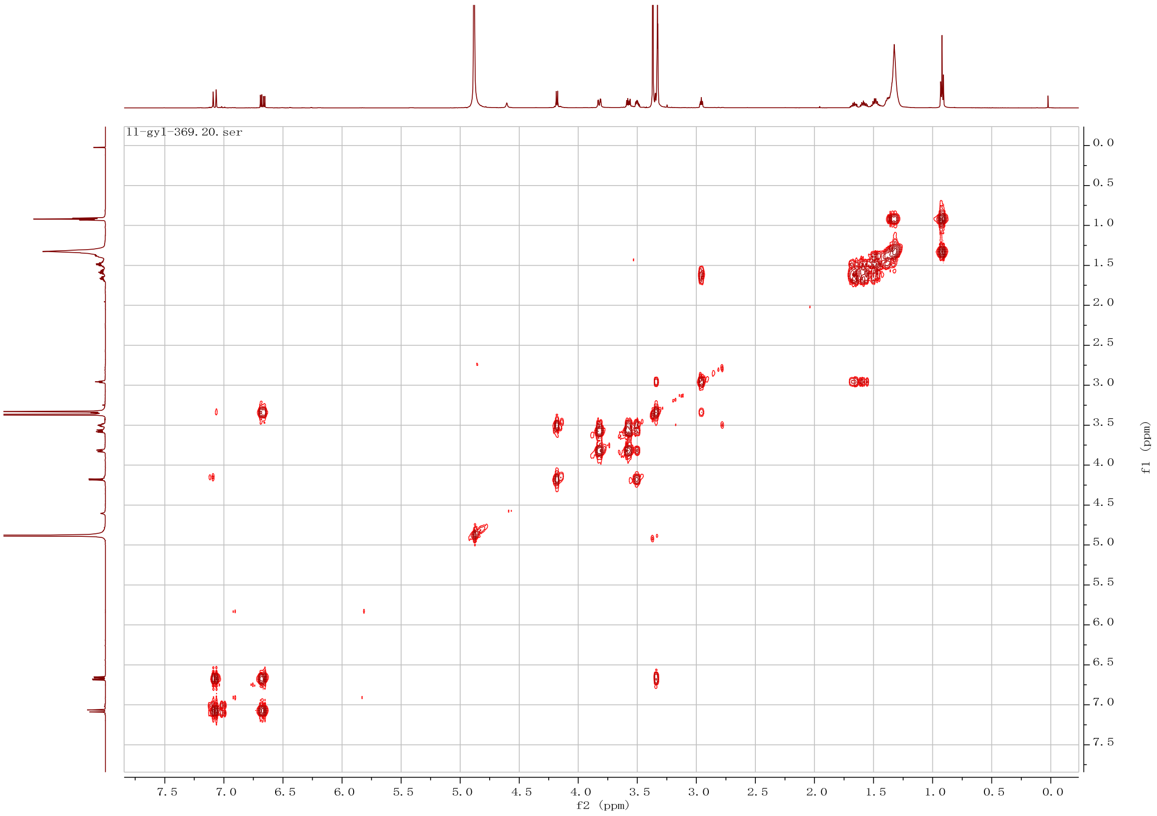

Figure S7. ^{1}H-^{13}C HSQC NMR spectrum of compound **1** in MeOD-d_4

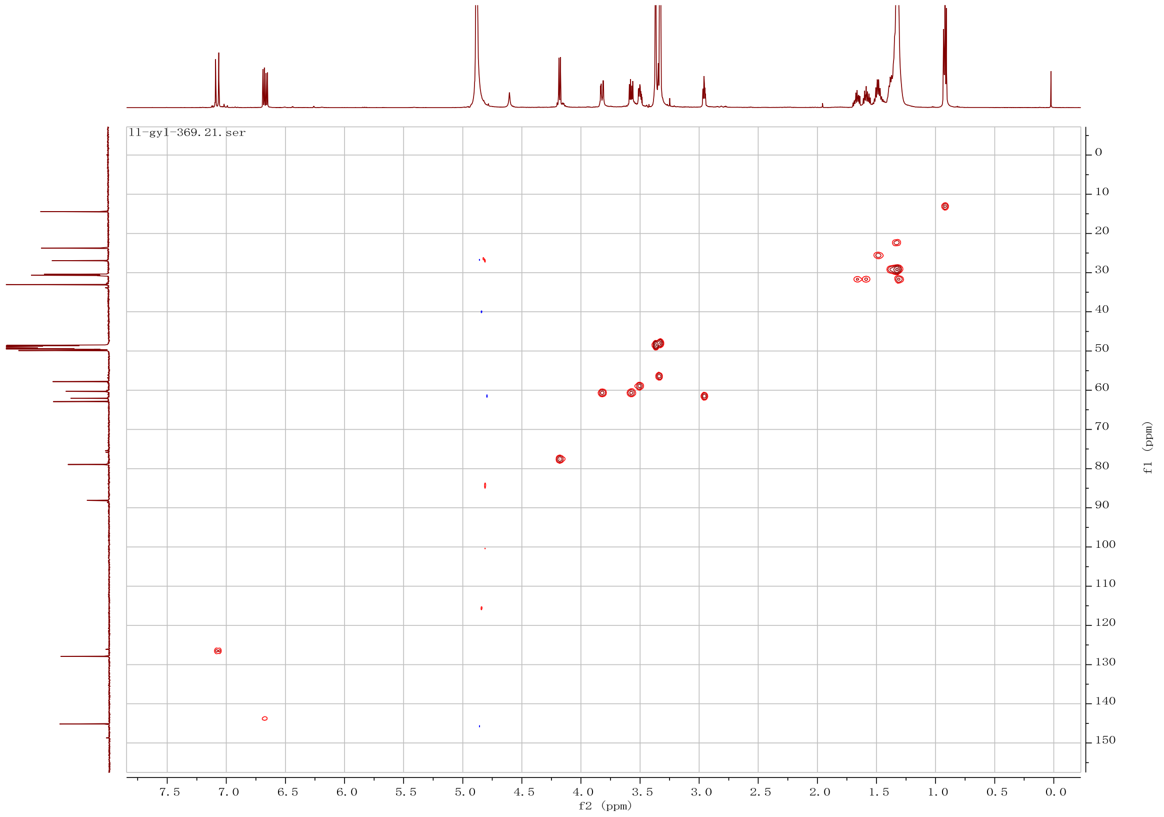

Figure S8. ^{1}H-^{13}C HMBC NMR spectrum of compound **1** in MeOD-d_4

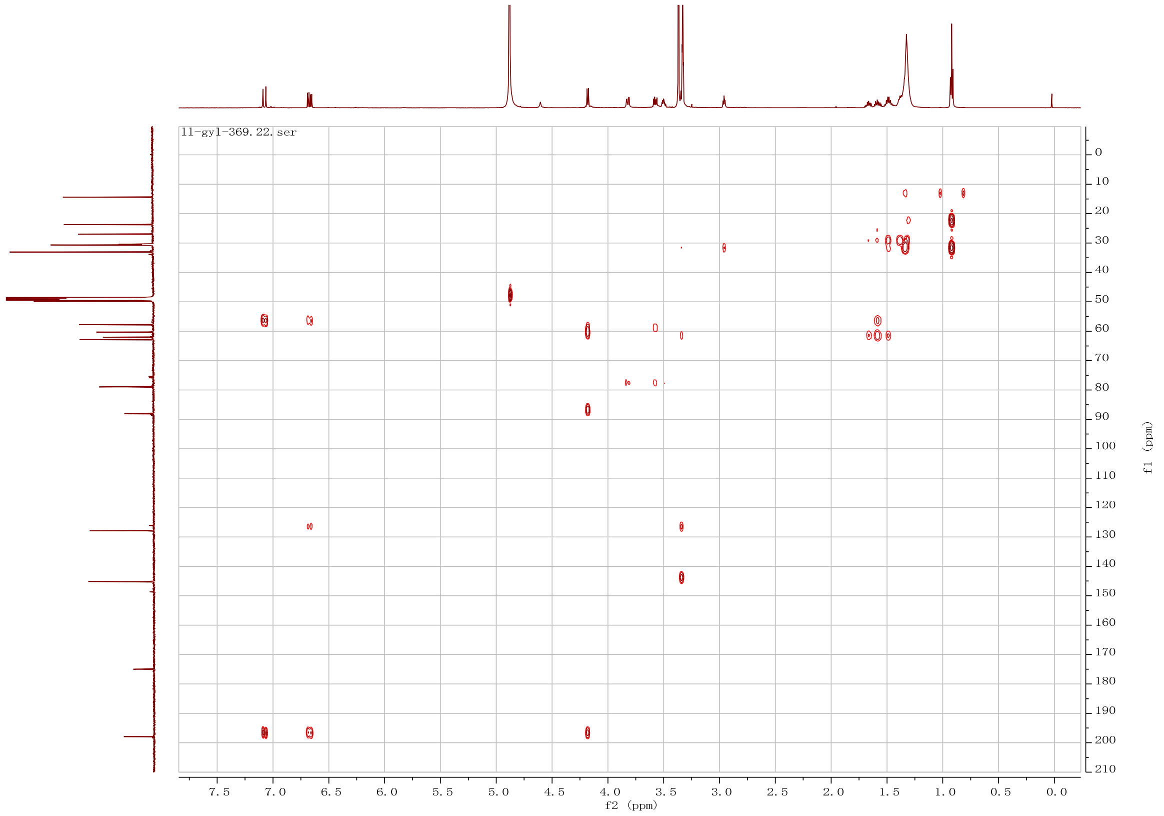

Figure S9. ^{1}H-^{1}H NOESY NMR spectrum of compound **1** in MeOD-d_4

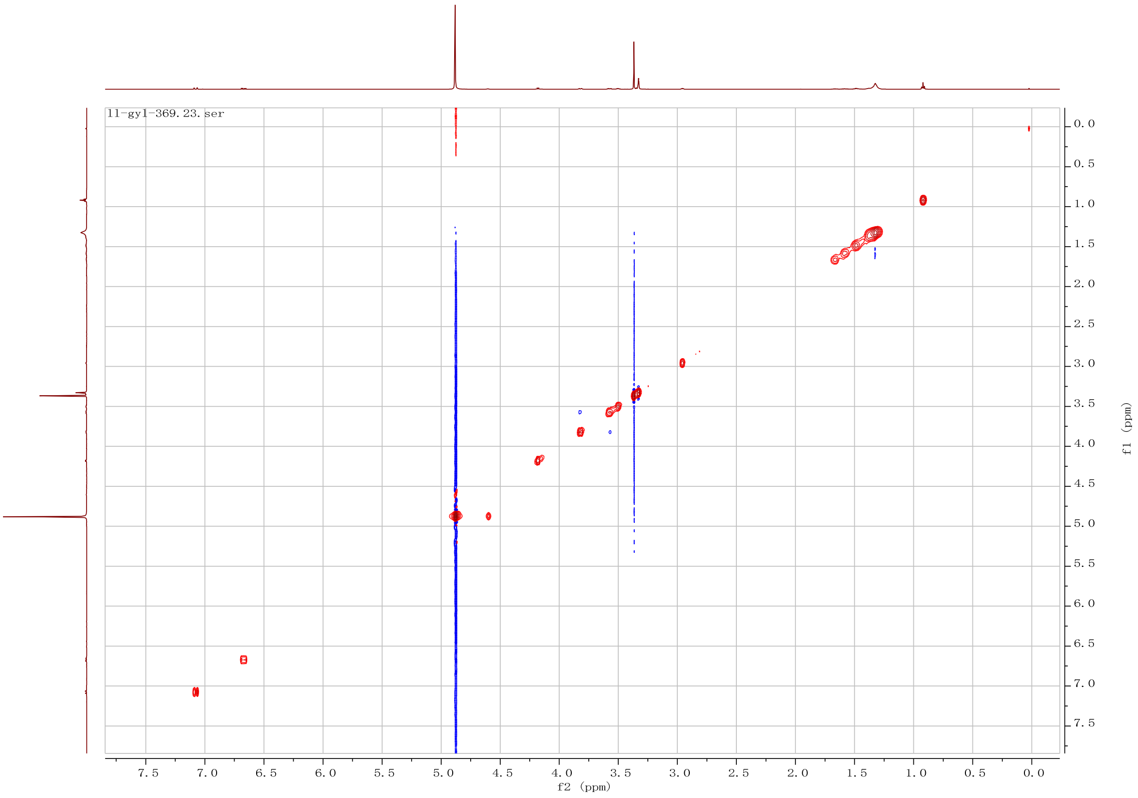

Figure S10. ^{1}H NMR spectrum (150 MHz) of compound **2** in MeOD-d_4

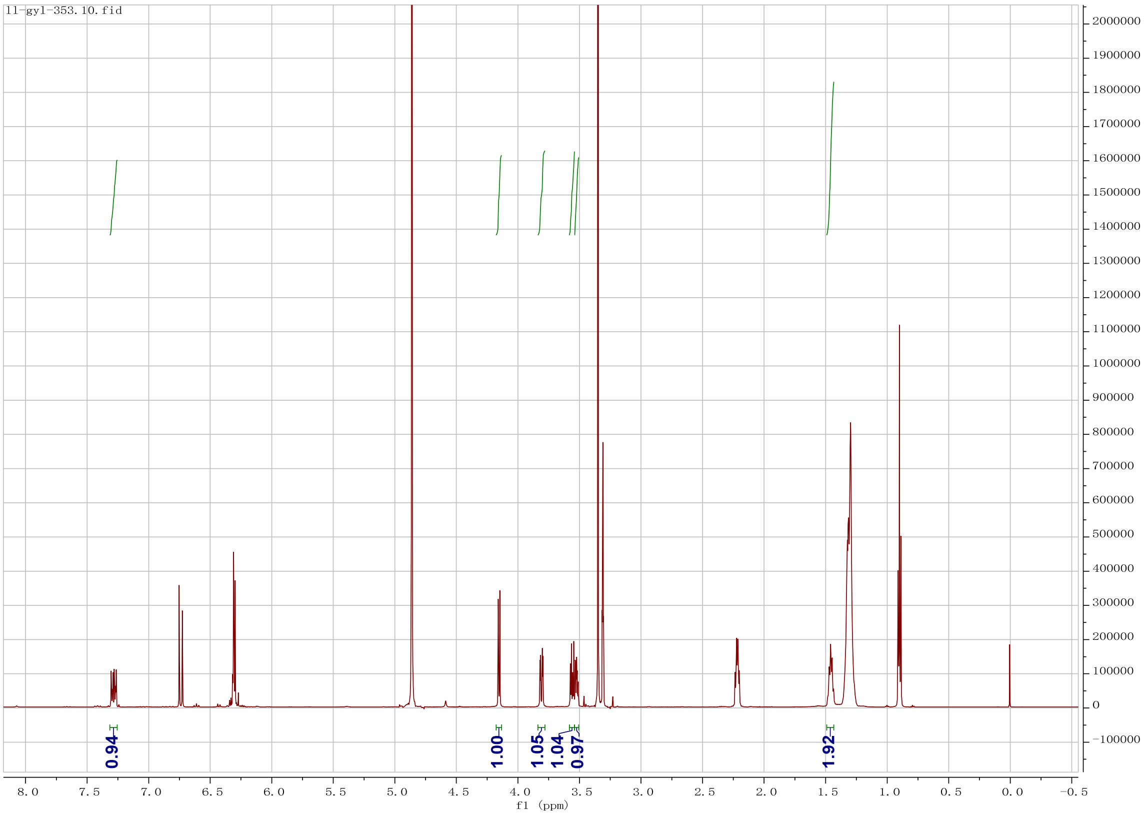

Figure S11. ^{13}C NMR spectrum (600 MHz) of compound **2** in MeOD-d_4.

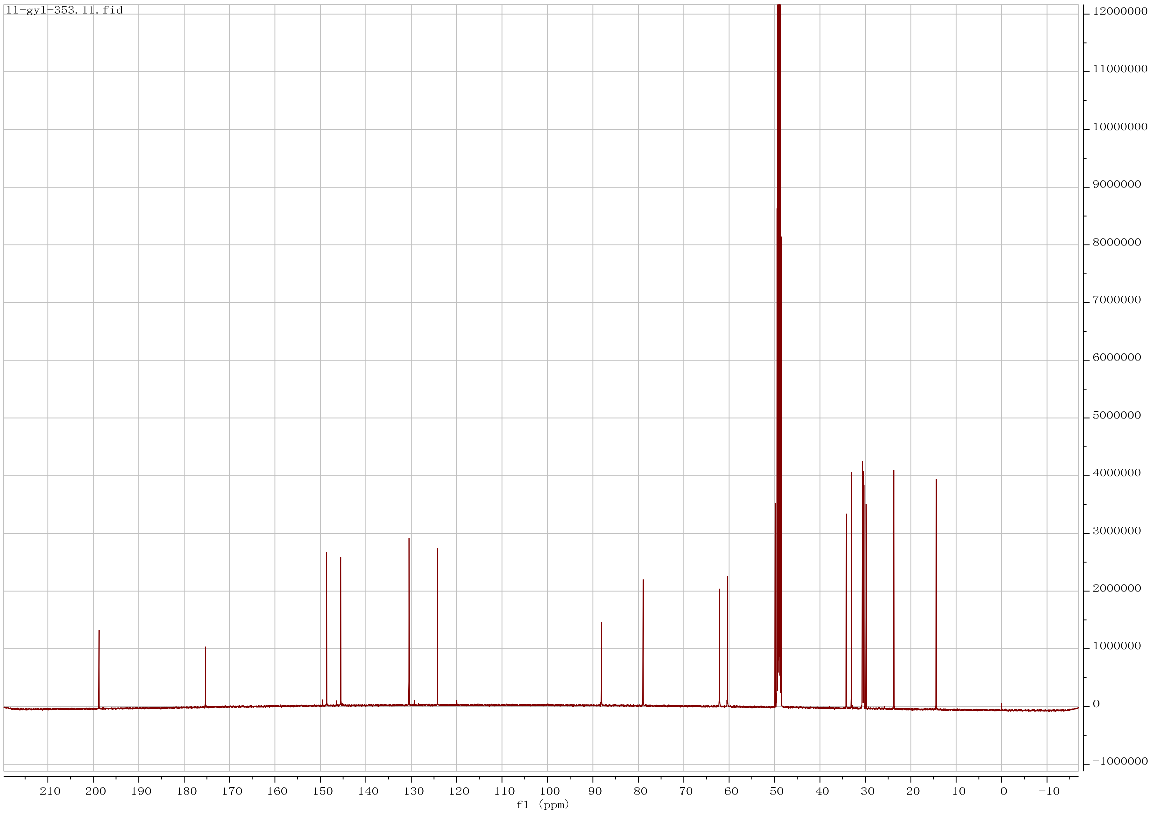

Figure S12. DEPT135° spectrum (150 MHz) of compound **2** in MeOD-d_4.

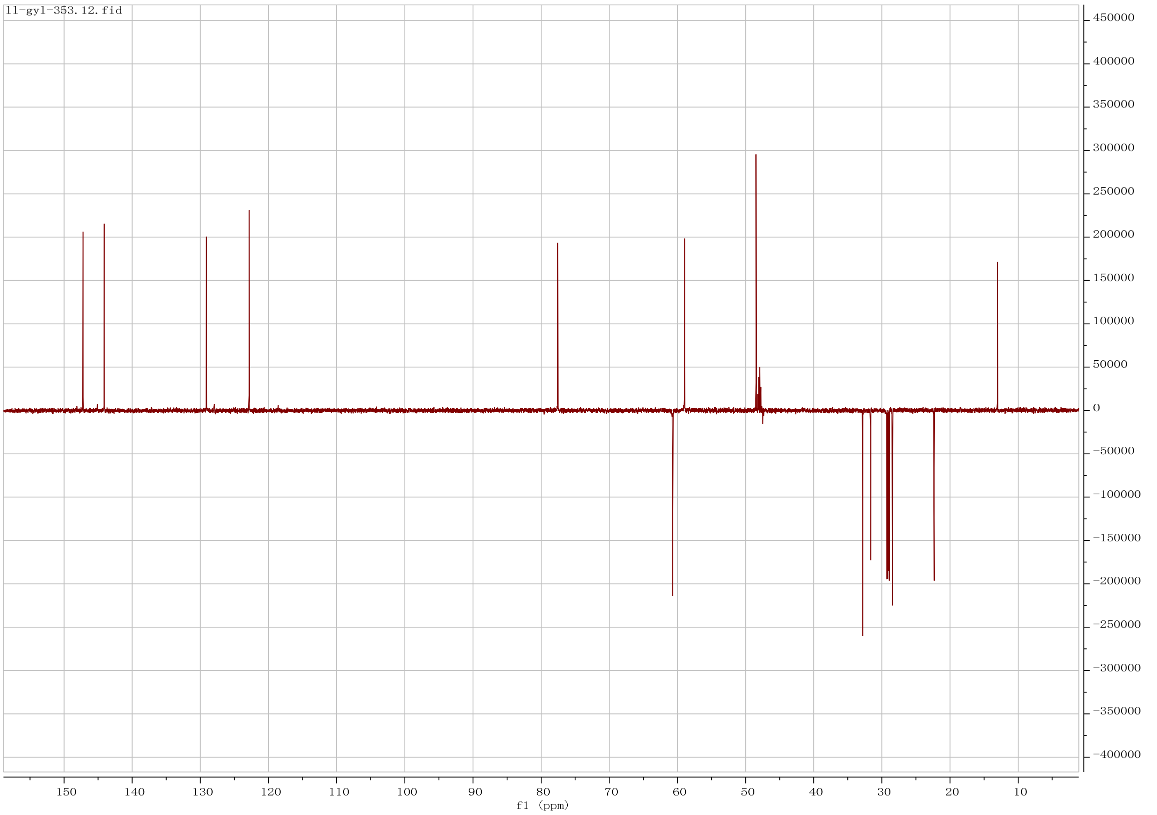

Figure S13. ^{1}H-^{1}H COSY NMR spectrum of compound **2** in MeOD-d_4

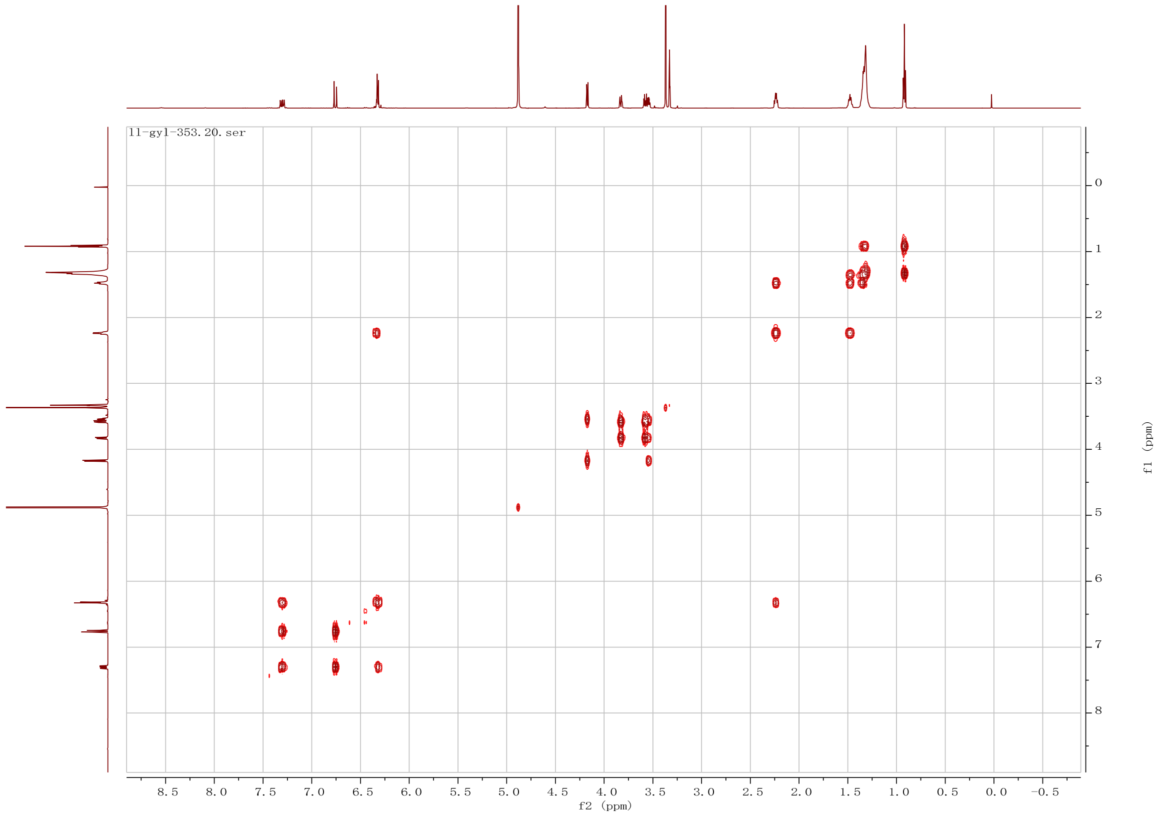

Figure S14. ^{1}H-^{13}C HSQC NMR spectrum of compound **2** in MeOD-d_4

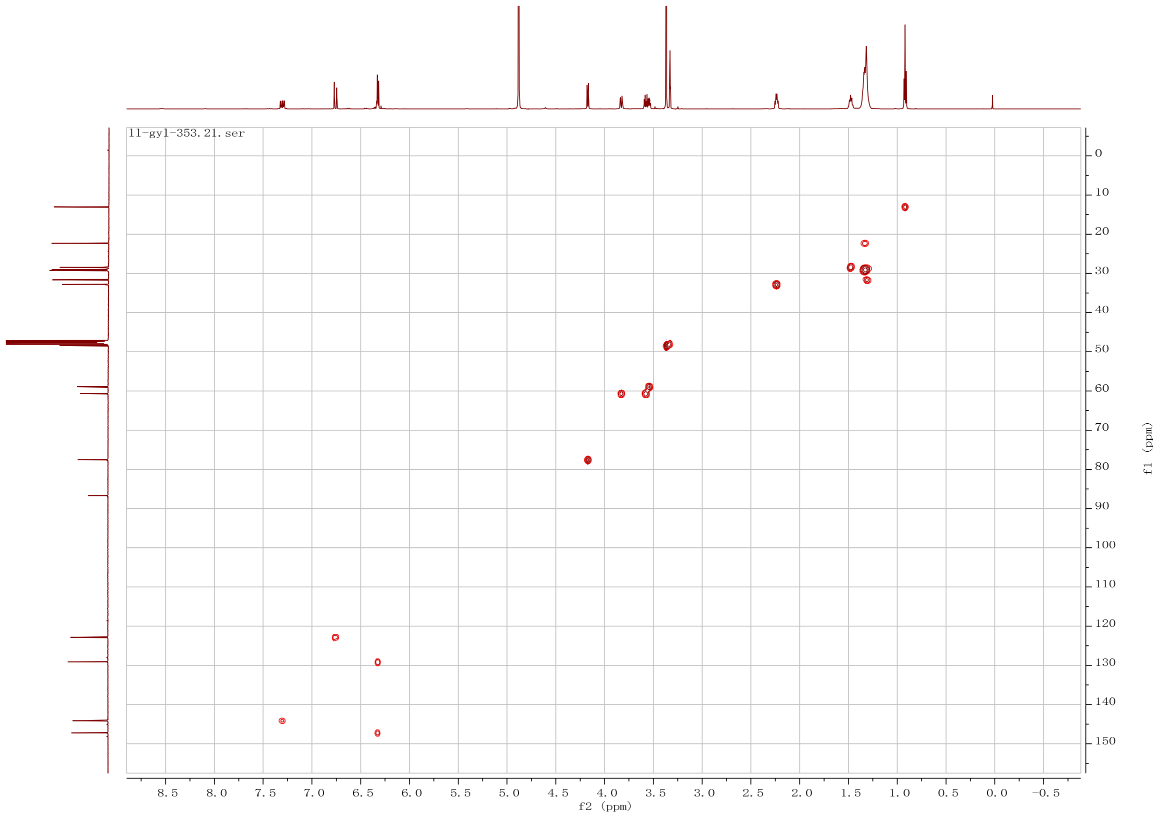

Figure S15. ^{1}H-^{13}C HMBC NMR spectrum of compound **2** in MeOD-d_4

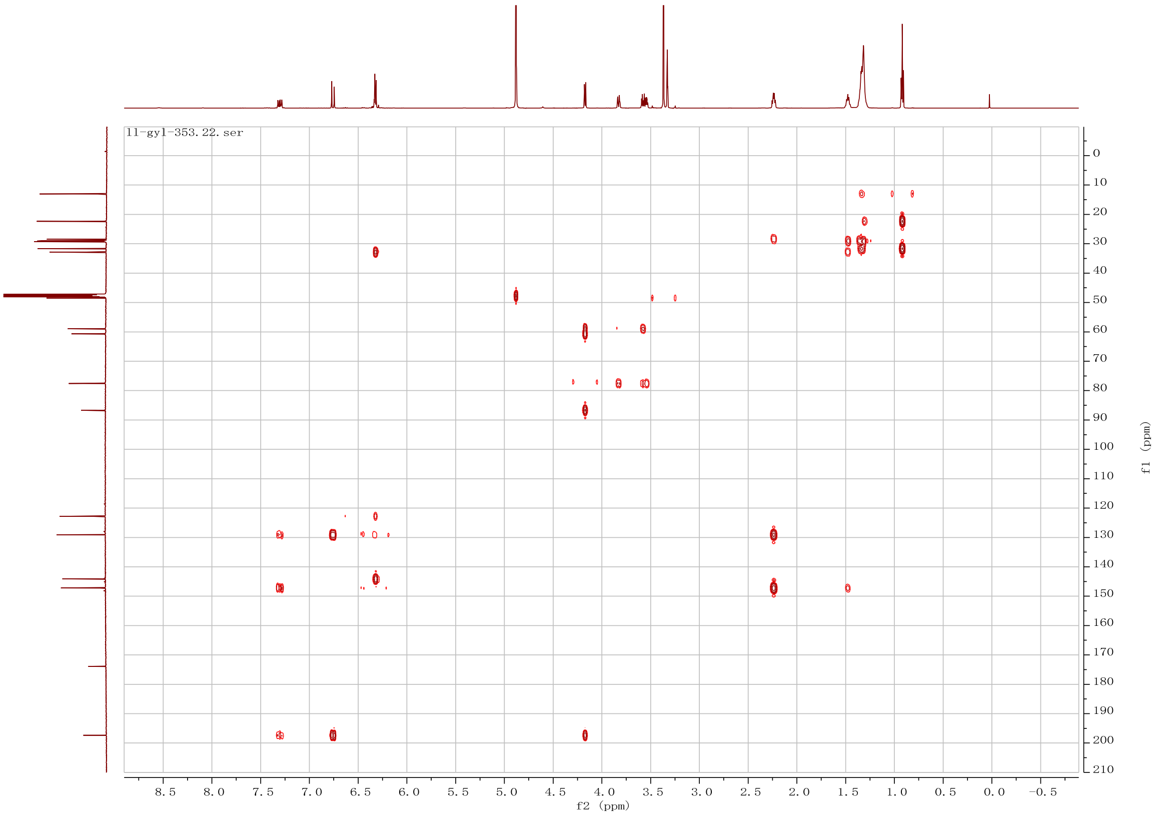

Figure S16. ^{1}H-^{1}H NOESY NMR spectrum of compound **2** in MeOD-d_4

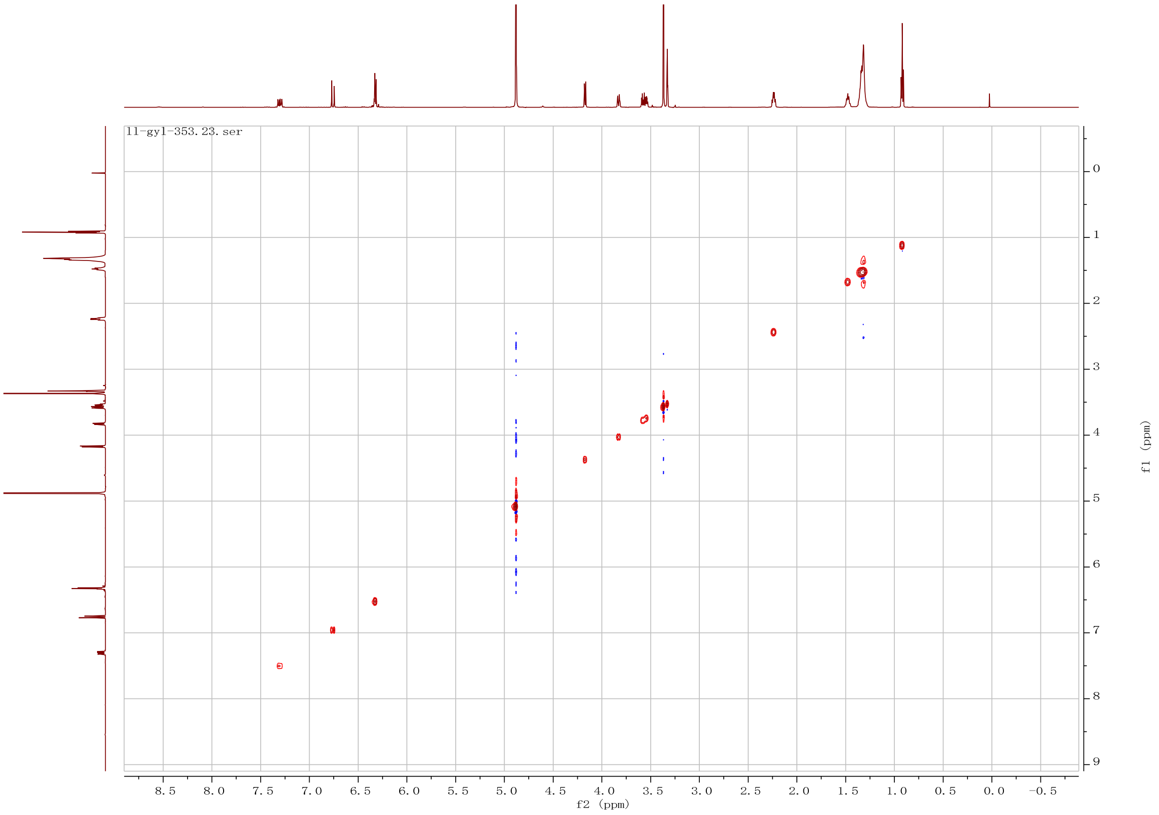

Figure S17. ^{1}H NMR spectrum (150 MHz) of compound **3** in DMSO-*d*6

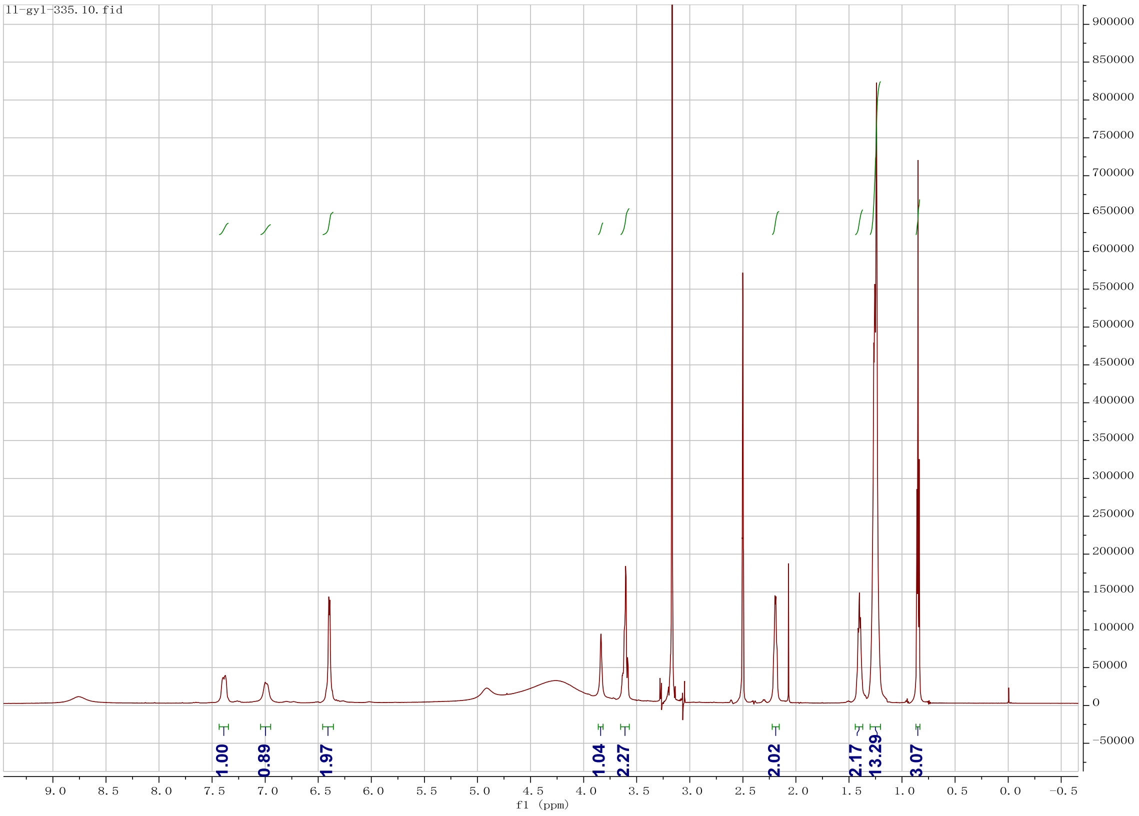

Figure S18. ^{13}C NMR spectrum (600 MHz) of compound **3** in DMSO-*d*6.

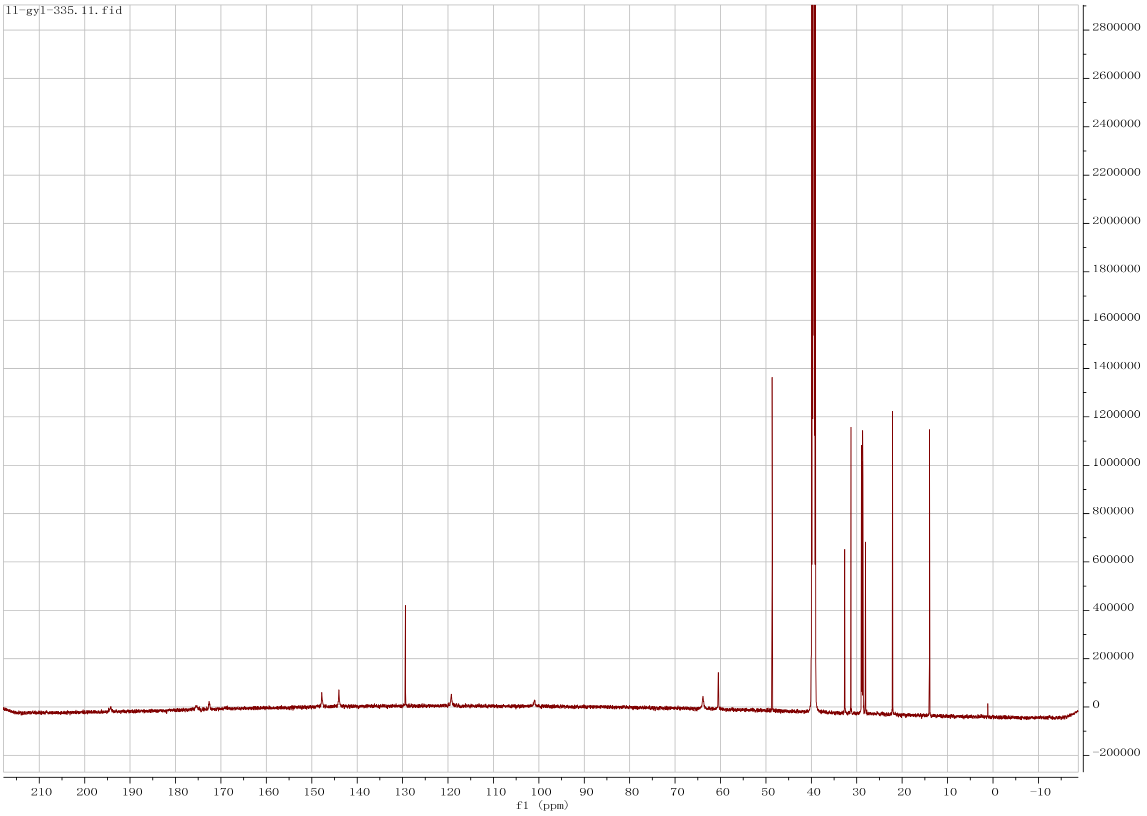

Figure S19. DEPT135° spectrum (150 MHz) of compound **3** in DMSO-d_6.

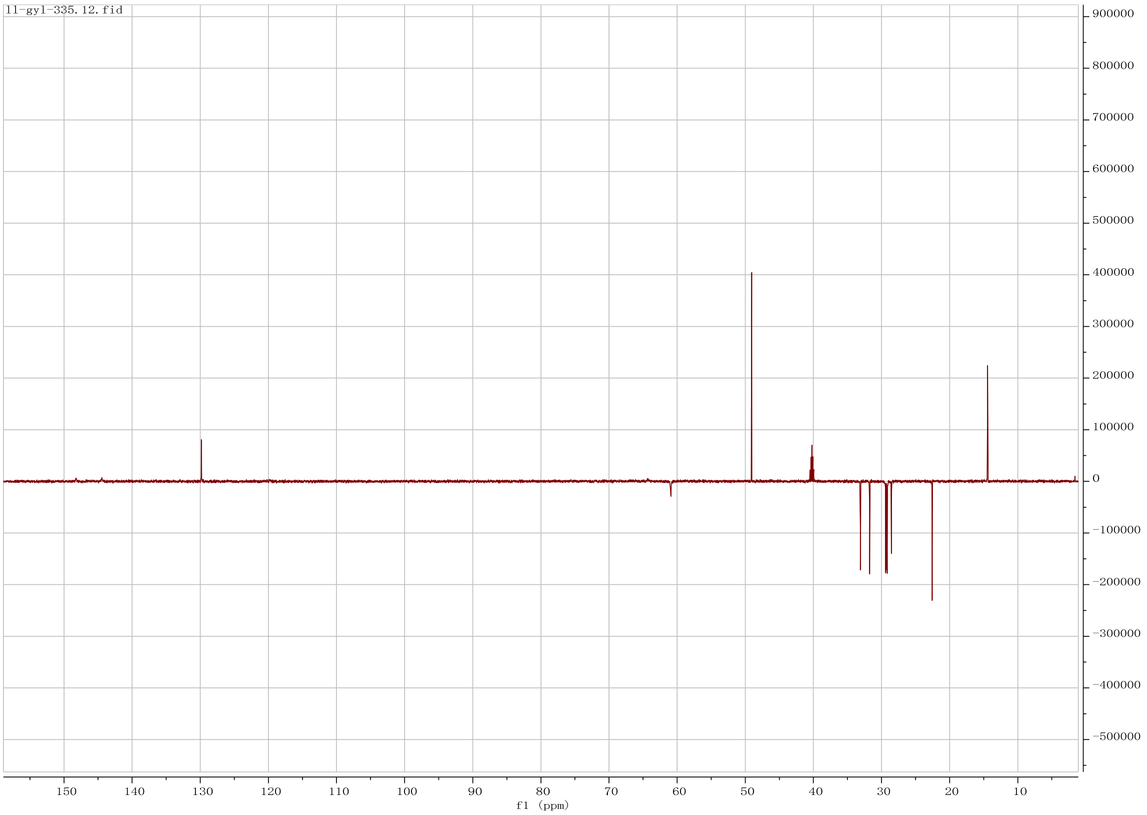

Figure S20. ^{1}H-^{1}H COSY NMR spectrum of compound **3** in DMSO-d_6

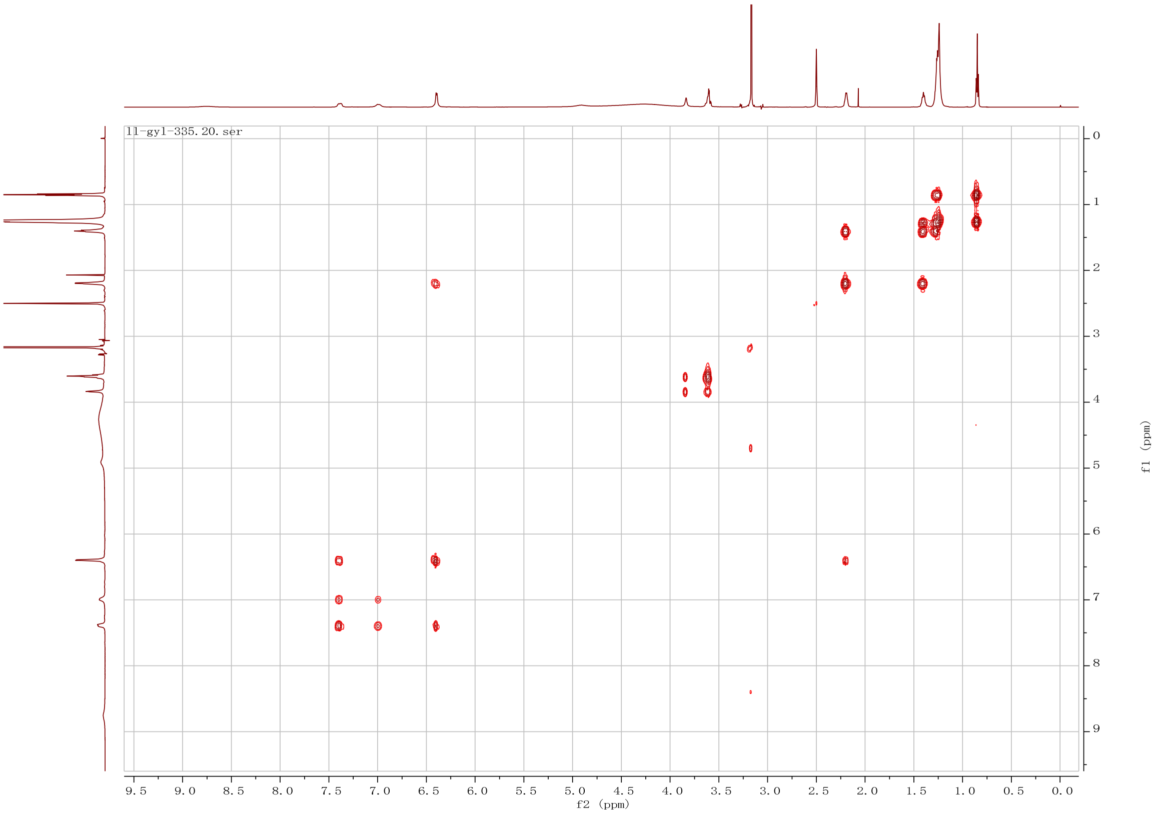

Figure S21. ^{1}H-^{13}C HSQC NMR spectrum of compound **3** in DMSO-d_6

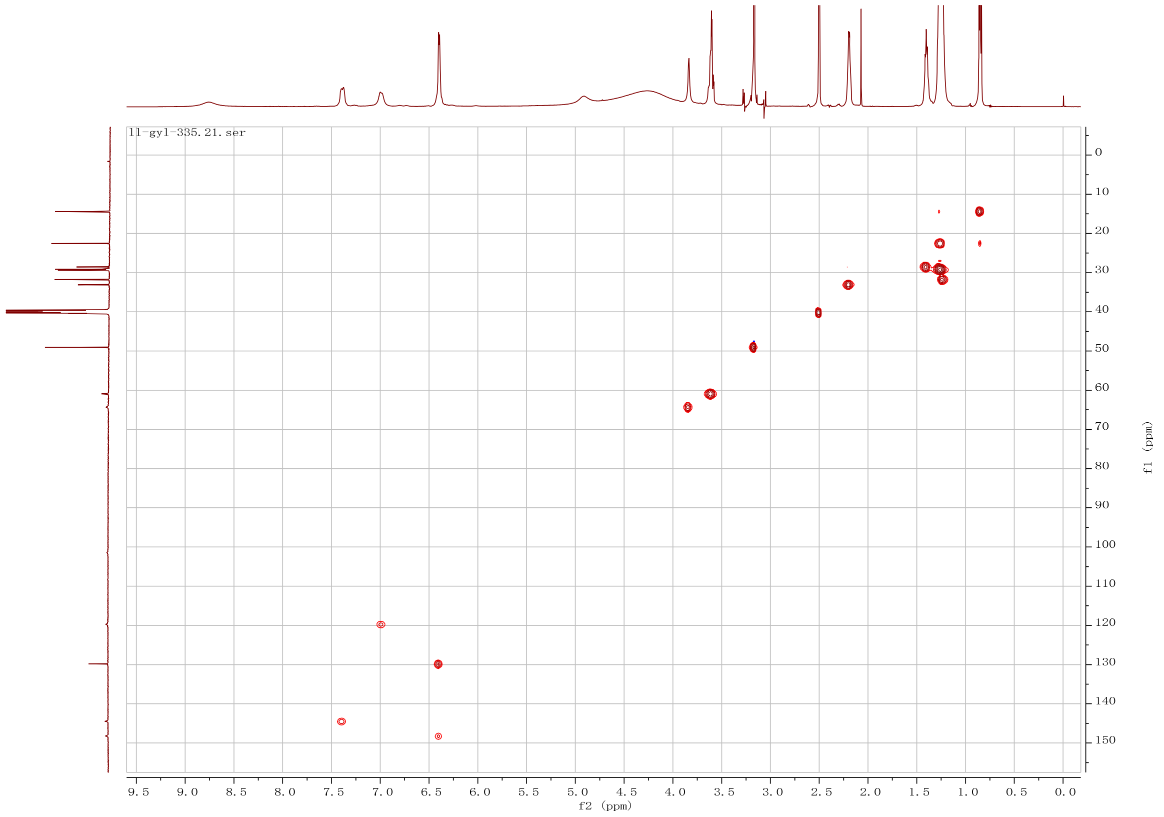

Figure S22. ^{1}H-^{13}C HMBC NMR spectrum of compound **3** in DMSO-d_6

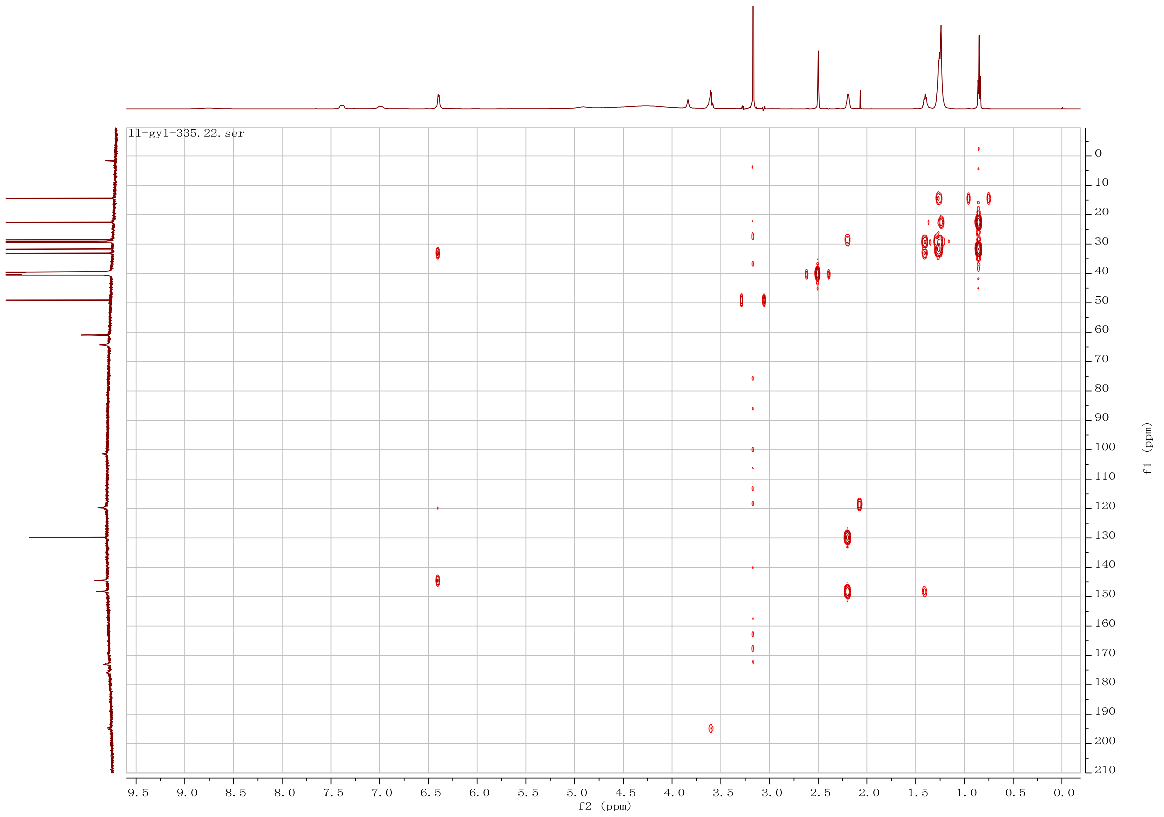

Figure S23. ^{1}H-^{1}H NOESY NMR spectrum of compound **3** in DMSO-d_6

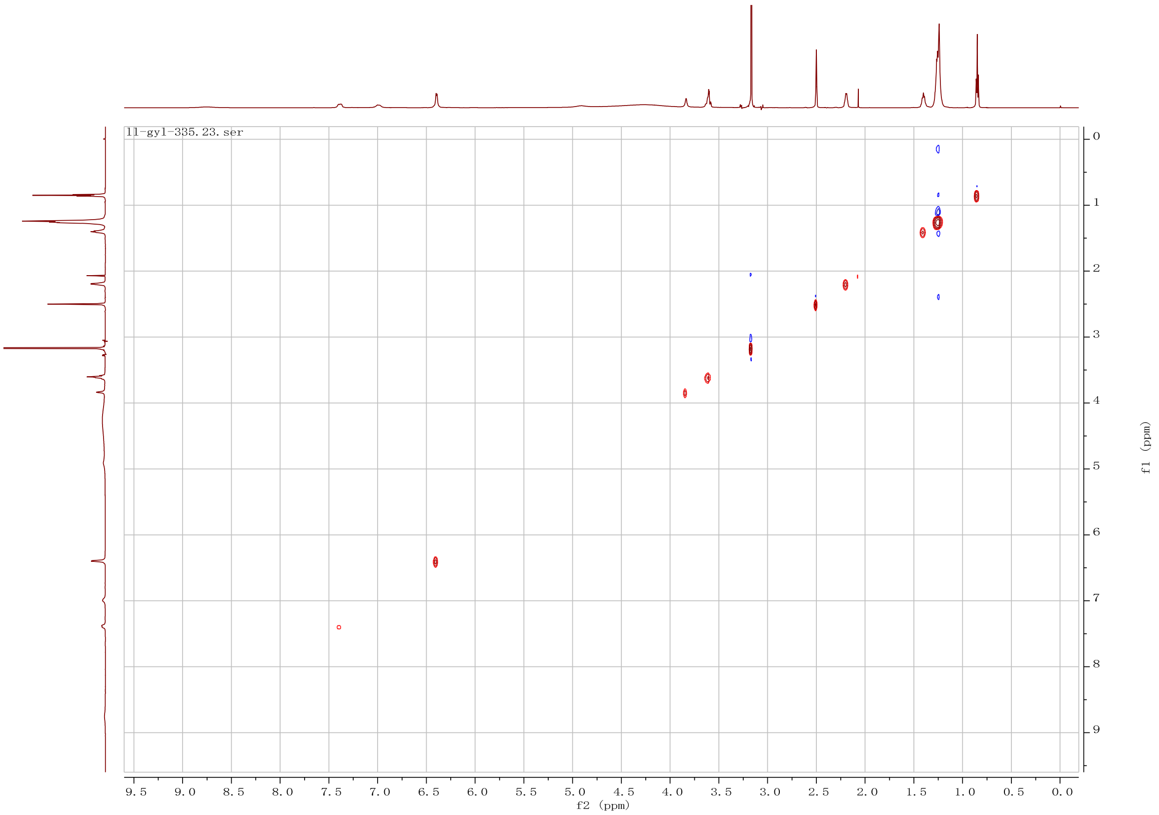

Reference

1. Schwartz, R.E.; Helms, G.L.; Bolessa, E.A.; Wilson, K.E.; Giacobbe, R.A.; Tkacz, J.S.; Bills, G.F.; Liesch, J.M.; Zink, D.L.; Curotto, J.E.; et al. Pramanicin, a novel antimicrobial agent from a fungal fermentation. *Tetrahedron* **1994**, *50*, 1675-1686, doi:https://doi.org/10.1016/S0040-4020(01)80843-7.
2. Harrison, P.H.M.; Duspara, P.A.; Jenkins, S.I.; Kassam, S.A.; Liscombe, D.K.; Hughes, D.W. The biosynthesis of pramanicin in Stagonospora sp. ATCC 74235: a modified acyltetramic acid. *Journal of the Chemical Society, Perkin Transactions 1* **2000**, 4390-4402, doi:10.1039/B006007K.
